# Modeling Red Blood Cell Deformation at Supraphysiological Strain Rates Using a Droplet Framework

**DOI:** 10.1007/s10439-026-04000-4

**Published:** 2026-01-29

**Authors:** Hannah P. Palahnuk, Nicolas A. Tobin, Keefe B. Manning

**Affiliations:** 1 Department of Biomedical Engineering, The Pennsylvania State University, University Park, State College, PA USA; 2 Applied Research Laboratory, The Pennsylvania State University, University Park, State College, PA USA; 3 Department of Surgery, Penn State College of Medicine, Hershey, PA USA

**Keywords:** Red blood cell, Erythrocyte, Deformation, Devices, Microfluidics, Hemolysis

## Abstract

**Purpose:**

Hemolysis remains a concern in mechanical circulatory support devices (MCSDs). Capturing flow-induced red blood cell (RBC) deformation is important to improve these technologies. Deformation models that are feasible for macroscale MCSD flows have not been calibrated with human RBC deformation data across multiple conditions. The purpose of this study is to modify and test a droplet deformation model that is applicable for MCSD flows for predicting human RBC deformation *in silico*.

**Methods:**

*In vitro* human RBC deformation is studied in microfluidic flows in two suspension viscosities (2.05 and 4.17 cP) at MCSD relevant strain rates (5,000 – 200,000 s^-1^ in shear flow; 330 – 13,160 s^-1^ in extensional flow). Modifications are made to the deformation model’s constitutive parameters to represent the observed RBC deformation *in silico*.

**Results:**

The calibrated model reproduces the unique RBC deformation behaviors observed in shear and extensional flows across a range of conditions. *In silico* shear deformation index data have mean absolute error (MAE) ≤ 0.15 compared to *in vitro* results for both viscosity conditions from 5,000 to 200,000 s^-1^. Peak *in silico* extensional deformation data demonstrate MAE ≤ 0.11 compared to our *in vitro* results for both viscosity conditions from 670 to 1,300 s^-1^, while MAE is higher (up to 0.17) for conditions at 330 s^-1^.

**Conclusion:**

The model adaptations successfully produce accurate RBC deformation results at MCSD relevant strain rates for two flow types and two suspension viscosities. The strengths of the model are in relatively high velocity gradient magnitudes and/or suspension viscosities where RBCs emulate liquid droplets.

**Supplementary Information:**

The online version contains supplementary material available at 10.1007/s10439-026-04000-4.

## Introduction

The human red blood cell (RBC) is a highly deformable biconcave disc-shaped formed element encapsulating a viscous hemoglobin solution and is responsible for oxygen delivery to all tissues [1]. The RBC’s hyperelastic cytoskeletal network and high surface area membrane allow it to withstand tremendous deformations throughout the circulatory system without permanent damage [1]. The RBC’s ability to deform is critical: a reduction in physiologic RBC deformability results in anemia, shortened RBC lifespan, and decreased capillary perfusion [2]. Decreased RBC deformability occurs in pathologies like malaria, diabetes, and sickle cell disease and after prolonged exposure to supraphysiological fluid stress [3–5]. RBCs can experience supraphysiological fluid stresses in blood-wetted medical devices, such as mechanical circulatory support devices (MCSDs) [6]. In these environments, high strain rates repeatedly and excessively deform RBCs, resulting in decreased deformability (RBC “stiffening”) and/or complete lysis of the membrane (“hemolysis”). Therefore, studying RBC deformation is important for understanding the cell’s mechanical behavior in disease and/or the potential for damage in medical devices.

Multiple *in silico* studies have investigated RBC deformation under static loads and/or flow conditions [7–13]. Typically, these studies model the membrane as an incompressible, viscoelastic shell and use tightly coupled numerical methods to capture deformation of a single or several RBC(s) [7–13]. While these studies provide valuable detail on the behavior(s) of one or a few cells under simple flow conditions, the computational cost of these numerical methods render the approaches inappropriate for application to the macroscale flows in medical devices. Computationally simpler approaches involve quantifying deformation and/or membrane areal strain via a strain-energy function or other empirically derived relationship, either of which typically involves using a scalar stress [14–16]. While these approaches demonstrate accuracy in canonical environments, they are problematic because a scalar stress does not appropriately represent the complex, multidimensional flow environment characteristic of MCSDs. Key information on the deformation state and multidimensionality of the flow is lost when using a scalar stress value. Deformation models are needed that (1) recapitulate the erythrocyte’s deformation accurately on the scale relevant to MCSDs and (2) do not use a scalar stress. Such models will aid in designing devices that do not excessively deform RBCs and/or lead to cell membrane damage and hemolysis.

A modeling framework that is computationally feasible for MCSD application is Maffettone and Minale’s droplet deformation model: a hyperbolic, transient evolution equation for a tensorial quantity that represents a 3-dimensional ellipsoid as it deforms in response to flow [17]. The model captures Eulerian droplet deformation using droplet properties and the 9-component velocity gradient tensor (i.e., no scalar stress), without the need for unrealistically expensive computational methods. This model is reasonable for RBCs, assuming they are neutrally buoyant liquid droplets that deform under applied flows and allowing their relaxation at flow cessation. The model has been applied to MCSD flow simulations to investigate device-induced hemolysis in multiple studies [18–23]. However, a major drawback of prior work is the lack of experimental validation of *in silico* droplet deformation, leaving open questions about the applicability to RBC deformation and hemolysis modeling.

Further, the conditions under which a flow-induced RBC deformation modeling framework are developed and validated are important. The RBC is known to deform differently across flow types, with up to 34 times greater deformation in extensional flows compared to shear flows at the same stress magnitude, highlighting the need to use multiple flow types in model development and validation studies [14, 16]. However, typical RBC deformation and/or mechanistic hemolysis models are developed only using shear flow regimes, while the flow in MCSDs is highly complex, with multiple flow types and aspects of turbulence [15, 24]. As one example, Chen and Sharp investigated hemolysis in hypodermic needles using a strain-based hemolysis model but used constitutive parameters on RBC strain mechanics only for shear flow [15]. A successful RBC deformation modeling approach should consider multiple flow types and flow conditions, including both shear and extensional flow regimes, to best tune numerical deformation equations.

Multiple *in vitro* studies have investigated flow-induced RBC deformation under different flow types and orders of magnitude in fluid strain [2, 14, 25–28]. These studies typically leverage microfluidic technologies to investigate RBC deformation and understand potential for damage in medical devices and to gain understanding in overall RBC rheological behavior(s). However, these studies use traditional microscopy techniques through transparent microfluidic channels, capturing only a 2-dimensional cell projection, which leaves a gap in understanding of 3-dimensional RBC deformation. Given the RBC’s unique biconcave disc shape and structure, a quantification of the deformation occurring in all three dimensions across a wide range of flow viscosities, types, and intensities is critical, especially when validating a flow-induced cell deformation modeling framework.

The focus of this study is to modify a droplet deformation model that is computationally feasible for application in flows characteristic of MCSDs such that it produces accurate deformation data compared to *in vitro* RBCs across a suite of conditions. Importantly, we make changes to the model’s constitutive deformation and relaxation parameters, calibrating them to best represent human RBC behavior. We expand on traditional microfluidic imaging techniques to capture multiple visualization planes of RBCs in shear and extensional flows *in vitro* to evaluate deformation in three dimensions. We complete these *in silico* and *in vitro* experiments in multiple MCSD relevant strain rates and flow types to show the breadth and applicability of the model. The numerical method presented here is a mechanistic, predictive model for human RBC deformation intended for use in MCSD flows.

## Materials and Methods

### In Vitro Experiments

***Working Fluids, Microfluidic Channels, and High-Speed Imaging Microscopy System:*** Following a Penn State Institutional Review Board approved protocol, whole blood was collected from healthy human adult volunteers in 6.0 mL plastic vacutainers (Beckton Dickinson, Franklin Lakes, NJ) with 3.2-wt.% sodium citrate solution (JT Baker, Phillipsburg, NJ). Donors abstained from taking steroids, nonsteroidal anti-inflammatory drugs, antibiotics, and allergy medications for 5 days prior to the blood draw. Blood was separated via density centrifugation at 300 g for 30 min, followed by 2700 g for 30 min to remove platelet-rich and platelet-poor plasma, respectively. Whole donor blood hematocrit (HCT) was measured using an Autocrit Ultra 3 (Becton Dickinson, Franklin Lakes, NJ). This study had 12 total blood donors (6 males and 6 females). The average donor HCT in this study (mean ± standard deviation) was 38.1 ± 2.5 %.

Isolated RBCs were resuspended at 0.5% HCT in Hanks balanced salt solution (Sigma-Aldrich, Burlington, MA) with 1% human serum albumin (Sigma-Aldrich, Burlington, MA). Albumin was used to retain the cells’ biconcave shape[29]. The working fluids were also supplemented with Dextran 40,000 Da (Spectrum Chemical, New Brunswick, NJ) at either 6.5 wt% or 13.5 wt% to study RBC deformation at two viscosity conditions: 2.05 cP and 4.17 cP, respectively. A viscosity of 4.17 cP was chosen to represent whole human blood (where the viscosity of the suspending solution is ~ 4.17 cP[30]). Further, as human blood is a suspension of formed elements and plasma, the effective fluid viscosity that deforming RBCs experience may be lower than that of whole blood. Therefore, 2.05 cP is used to capture a lower viscosity condition. The blood suspensions were used within 5 hours of the blood draw and were stored in polypropylene conical tubes prior to use.

Table 1 lists the viscosities, which were confirmed using a Vilastic-3 Viscoelasticity Analyzer (Vilastic Scientific Inc., Austin, TX), along with the pH and osmolarity of the working fluids, which were confirmed to be within physiological range. The pH was measured using a SevenEasy pH meter (Mettler Toledo, Columbus, OH), and the osmolarity was measured using a freezing point osmometer (Advanced Instruments, Inc., Norwood, MA).

**Table 1:** Suspension viscosity, density, pH, and osmolarity for *in vitro* deformation experiments. All suspensions were 0.5% HCT. Data are reported as mean ± standard deviation

Fluid viscosity (cP)	Weight % dextran 40 kDa	Fluid density (g/mL)	pH	Osmolarity (mOsm/kg H_2_O)
4.17 ± 0.04	13.5	1.01 ± 0.004	7.29 ± 0.18	321
2.05 ± 0.02	6.5	1.0 ± 0.01	7.21 $\pm$ 0.08	294

**Fig. 1:**
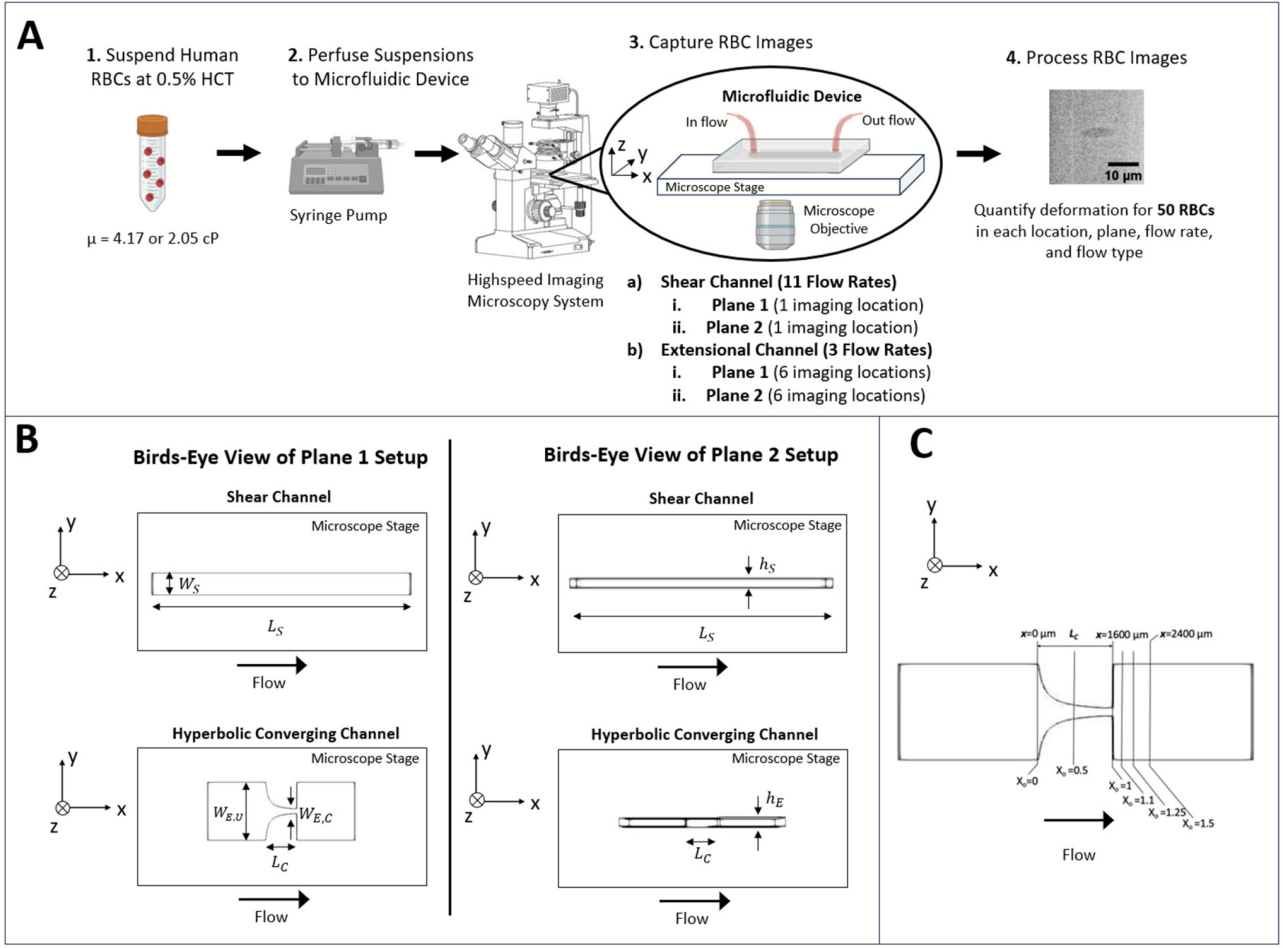
Workflow and imaging platform for *in vitro* RBC deformation experiments. **A** Workflow for collecting and quantifying *in vitro* RBC deformation data using a high-speed imaging microscopy system. Dilute (0.5% HCT) RBC suspensions were perfused through the microfluidic channels mounted on the microscope using a syringe pump. The microscope was connected to a high-speed camera to capture cell images. Channels were oriented in either the Plane 1 or Plane 2 visualization setup; images were collected for 11 flow rates in shear flow and 3 flow rates in extensional flow. Images were collected in one location in the shear channel and in 6 locations in the hyperbolic converging channels. The RBC images were then processed, and deformation quantified for 50 RBCs in each imaging plane (Plane 1, Plane 2) for both viscosity conditions (4.17 and 2.05 cP), both flow types (shear, extension), all flow rates, and all imaging locations. **B** Bird’s-eye view of both visualization planes, showing both the shear and hyperbolic converging channels and the flow direction with a thick black arrow. In Plane 1, the length and width dimensions of the channels are both parallel to the microscope stage. In Plane 2, the channel is rotated 90°, and the length and height of the channel are parallel to the microscope stage. *L*_*S*_ = 1.25 cm, *W*_*S*_ = 400 μm, *W*_*E,U*_* = *2000 μm, *W*_*E,C*_* = *150 μm, *L*_*C*_ = 1600 μm, *h*_*S*_ = 50 μm, and *h*_*E*_ = 35 μm. **C** Normalized axial position in the hyperbolic converging microchannel. The normalized axial position, X_o_, is equivalent the axial distance (*x*) of the image location from the start of the contraction (*x* = 0) divided by the length of the contraction, *L*_*c*_. Images were collected at 6 locations throughout the channel: X_o_ = 0, 0.5, 1, 1.1, 1.25, and 1.5. (Note: coordinate axes are provided for spatial orientation in each panel of the figure)

For all experiments, the RBC solutions were perfused through microfluidic channels mounted on an Olympus IX71 (Olympus, Shinkuku City, Tokyo, Japan) inverted microscope stage using a syringe pump (KD Scientific, Holliston, MA) (Fig. 1A). A simple shear microchannel and a hyperbolic converging microchannel (Fig. 1B) were used to produce shear and extensional flows, respectively. The hyperbolic converging channel produces a nearly constant extensional fluid strain rate along the centerline of its contraction length (*L*_*c*_, Fig. 1B) [14, 26]. Table 2 details the flow conditions used in this study, which represent a wide range to accommodate MCSD flows. Using Eq. 1, the wall shear rate $(\dot{\gamma }_{{{\mathrm{Shear}}}} )$ in the shear channel is calculated, where *Q* is the flow rate, and ${W}_{S}$ and ${h}_{S}$ are the channel width and height, respectively (Table 2, Fig. 1A). Using Eq. 2, the average extensional strain rate ($\dot{\gamma }_{{{\mathrm{Extension}}}} )$ along the hyperbolic contracting channel’s centerline is calculated, where *W*_*E,U*_ is the upstream width of the channel, *W*_*E,C*_ is the width of the channel throat, *L*_*c*_ is the length of the channel contraction, and *h*_*E*_ is the channel height as shown in Fig. 1B. The extensional strain rate along the centerline of the channel reaches its maximum value (~2 $*\dot{\gamma }_{{{\mathrm{Extension}}}} {)}$ at approximately 0.5*L_*c*_ and remains constant until the end of the contraction (Table 2).

1 $$\dot{\gamma }_{{{\mathrm{Shear}}}} { } = \frac{6Q}{{W_{S} h_{S}^{2} }}$$

2 $$\dot{\gamma }_{{{\mathrm{Extension}}}} = \frac{Q}{{L_{C} h_{E} }}\left( {\frac{1}{{W_{E,C} }} - \frac{1}{{W_{E,U} }}} \right)$$

**Table 2:** Flow conditions used for shear and extensional flow experiments. The wall shear rate $(\dot{\gamma }_{{{\mathrm{Shear}}}} )$ ranges from 5,000 to 200,000 s^-1^ and maximum extensional strain rate (~2*$\dot{\gamma }_{{{\mathrm{Extension}}}}$) ranges from 330 to 1,330 s^-1^ using the flow rates listed

Shear flow		Extensional flow	
Flow rate,* Q* (mL/min)	Wall shear rate, ${\dot{\gamma }}_{Shear}$ (s^-1^)	Flow rate, *Q* (mL/min)	Extensional strain rate, ~ 2*$\dot{\gamma }_{{{\mathrm{Extension}}}}$ (s^-1^)
0.05	5,000	0.1	330
0.075	7,500	0.2	670
0.1	10,000	0.4	1,330
0.15	15,000		
0.2	20,000		
0.3	30,000		
0.5	50,000		
0.7	70,000		
1.0	100,000		
1.5	150,000		
2.0	200,000		

To capture three-dimensional RBC deformation data under flow, two microfluidic channel orientations were used in the microscopy imaging system (Fig. 1A, B). For the first orientation, channels were oriented with their length dimension (*L*_*S*_ or* L*_*C*_) and width dimension (*W*_*S*_ or *W*_*E,U*_*/W*_*E,C*_) parallel to the microscope stage (“Plane 1”) such that the vorticity plane was visualized (Fig. 1B). The channel height (*h*_*S*_ or *h*_*E*_) is normal to the microscope stage in Plane 1. For the second orientation, the channels were rotated 90° and mounted with their length (*L*_*S*_ or* L*_*C*_) and height (*h*_*S*_ or *h*_*E*_) parallel to the microscope stage (Fig. 1B) (“Plane 2”). The channel width (*W*_*S*_ or *W*_*E,U*_*/W*_*E,C*_) is normal to the microscope stage in Plane 2. These two set-ups allowed image acquisition of all three dimensions of the deformed RBCs.

For Plane 1 and Plane 2 in shear flow, the RBCs were imaged at the channel wall for the highest shear rate (*z* = 50 μm in Plane 1*, y* = 50 μm in Plane 2) and at *x* = 0.5*L_*s*_ to ensure fully developed flow (Fig. 1A, B). The cell-free layer (CFL) in the shear channels was small in this study: the CFL measured ~ 3 µm in the *z*- and *y*-directions in Planes 1 and 2, respectively. This migration affected the applied shear rate by only 5.6% and is therefore assumed to be negligible. For both visualization planes in extensional flow, RBCs were imaged at 6 discrete locations to evaluate deformation as cells traversed the converging region and sudden expansion, with positioning denoted by X_o_ (Fig. 1A, C). X_o_ represents an axial position normalized to the length of the contraction (*L*_*C*_). The locations imaged were at X_o_ = 0, 0.5, 1, 1.1, 1.25, and 1.5 (Fig. 1A, C). These locations were chosen to capture the RBC deformation behavior as an RBC began to extend (X_o_ = 0, 0.5), at peak extension in the channel throat (X_o_ = 1), and as an RBC relaxed in the channel’s sudden expansion (X_o_ = 1.1 – 1.5). Three locations were imaged downstream of the channel throat as benchmark simulations showed this region has high spatial variability in the velocity gradient tensor. All locations imaged in the hyperbolic converging channels were taken at the center plane of the channel (*z* = 17.5 μm in Plane 1, *y* = 17.5 μm in Plane 2) to capture purely extensional flow (Fig. 1B).

Still frame images of flowing RBCs were captured with a PCO-1600 high-speed camera for each condition (Fig. 1A). The camera exposure time used (3 μs) was low to minimize blur as the RBCs flowed through the channels. All flow conditions listed in Table 2 were tested *in vitro* for both viscosity conditions and both visualization planes and were repeated 6 times (*n* = 6). For all imaging experiments in Plane 1 and the shear experiments in Plane 2, a 60xW/1.2 UPlanApo objective (Olympus, Shinkuku City, Tokyo, Japan) (resolution ~ 8.2 pixels/$\upmu$m) was used to visualize the RBCs. For extensional flow experiments in Plane 2, a longer working distance was required to reach the required depths into the channel, and thus a 40x/0.6 LUCPlanFl objective was used (Olympus, Shinkuku City, Tokyo, Japan) (resolution ~ 5.4 pixels/$\upmu$m).

***Channel Fabrication:*** Shear and extensional channels used in the Plane 1 experiments and the shear channels for Plane 2 experiments were constructed from polydimethylsiloxane (PDMS). Briefly, SU-8 wafers were manufactured in Penn State’s nanofabrication facility using the geometries shown in Fig. 1B (dimensional uncertainty $\pm$ 0.3 μm). The feature on the master SU-8 wafers for the shear channels used in Plane 2 were rotated 90° from those in Plane 1 to produce the same chip geometry in the Plane 2 orientation. To make the PDMS channels, silicone (Ellsworth Adhesives, Irondequoit, NY) was combined 1:10 base:curing agent and cured at 60° C for 4 hours on the wafer. After the channels were cured, they were removed from the master, rinsed with 70% EtOH and sealed to coverslips using a plasma cleaner (Harrick Plasma, Ithaca, NY) at 18 W and ~ 550 mTorr for 35 s. Because of the complex geometry of the hyperbolic converging channel, a custom fixture was fabricated for Plane 2 experiments (Potomoc Photonics, Baltimore, MD) by micro-CNC milling the channel geometry into polymethyl methacrylate in the appropriate orientation. Before experiments, all channels were coated with 1-wt% bovine serum albumin (Sigma-Aldrich, Burlington, MA) for 1 h to decrease non-specific adhesion of RBCs to the channel and/or glass during experiments.

**Fig. 2:**
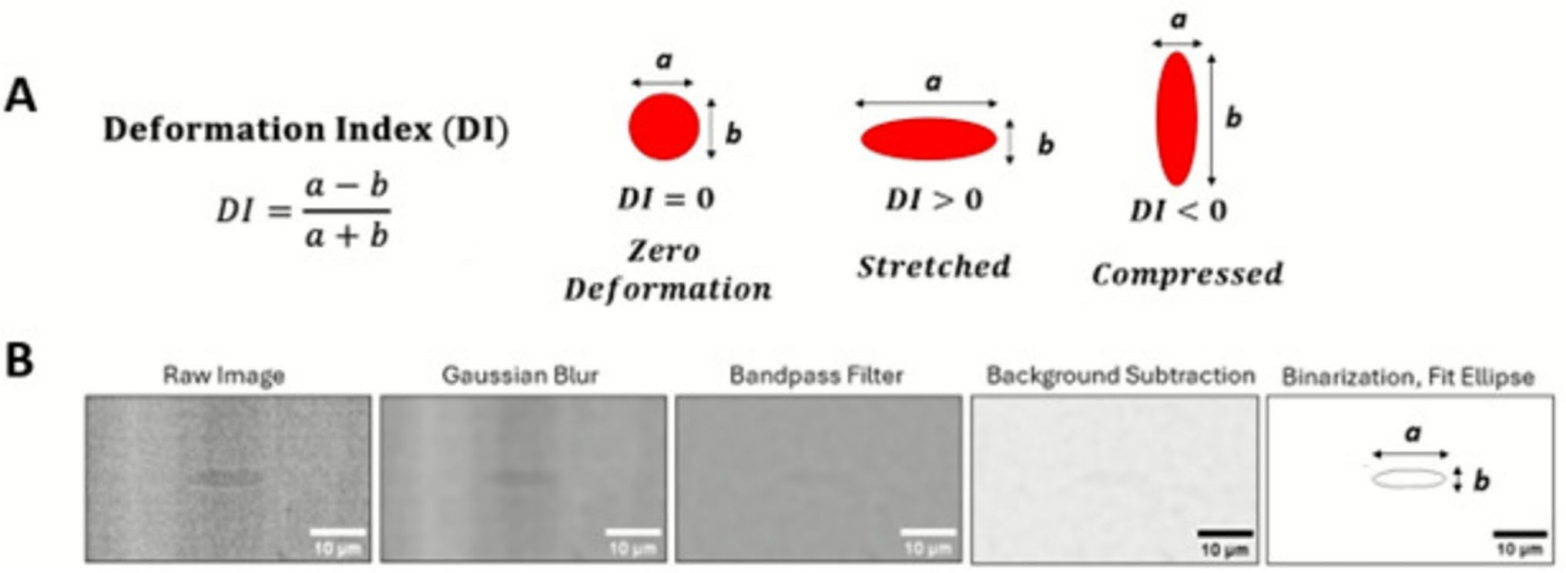
Visual representation of the deformation index (DI) used to quantify flow-induced RBC deformation and image processing workflow. **A** Deformation index equation using the major and minor axes of the deformed RBCs. The magnitude and sign of the DI show the relative intensity and type of deformation experienced by the cells. Positive DI is a stretched cell, while negative is a compressed cell. **B** Visual representation of the image processing code workflow. The raw images were smoothed and a Gaussian filter is applied first to blur the images. Next, a bandpass filter and background subtraction were applied to remove background noise. The image was then binarized and ellipses fit to the cell contour. The major and minor axes of the ellipses of the cell contour were used for the DI calculation (*a, b*)

***Image Processing and Experimental Error Quantification****:* The RBCs’ deformation was quantified for 50 cells in each experimental condition. This resulted in 300 total RBCs in each visualization plane (Plane 1, Plane 2) for each condition and channel location across the *n* = 6 experiments. Images were processed with in-house codes in FIJI (“FIJI is just ImageJ,” National Institutes of Health), and deformation was quantified with a deformation index (DI, Fig. 2A). Briefly, images were smoothed, Gaussian filtered, and binarized, and ellipses were fit to the major and minor axes of the captured cells to quantify DI (Fig. 2B). The sign of the DI describes the environment in which the cell is deformed (stretched or compressed) within the flow (Fig. 2A).

The error produced in image acquisition and image processing was quantified by imaging microspheres (Thermo Fisher Scientific, Waltham, MA) flowing through both the shear and hyperbolic converging channels. The microspheres (5.02 ± 0.03 µm diameter) were seeded at 1.5 wt% in 1x phosphate-buffered saline, and images were acquired using the methods previously described. These experiments served as a verification that there is minimal error in the imaging technique and processing workflow. The particle diameter was quantified using the image processing code for all acquired images of the flowing particles. For all shear flow conditions, the error range on quantified particle diameter was between 5.7 and 6.7%. For the extensional flow conditions, the error quantified on the particle diameter was 4.6–10.4% from the lowest to highest flow conditions, respectively. These particle experiments demonstrated there is minimal error using the imaging technique (with a 3-μs exposure time) and image processing workflow at the flow conditions in this study.

***Micro-Particle Image Velocimetry (***${\boldsymbol{\mu}}$***PIV) flow visualization:*** Flow was quantified using a $\upmu$PIV system (TSI Inc., Shoreview, MN) to validate *in silico* fluid mechanics results. Briefly, channels were mounted on a Nikon Eclipse TE300 inverted fluorescent microscope stage (Nikon, Melville NY) and perfused with a solution of deionized water and 2-wt.% 1 $\upmu$m diameter polystyrene fluorescent microspheres (540/560 nm excitation/emission). Two Nd:Yag 532-nm laser pulses, controlled by a LaserPulse Synchronizer (TSI Inc., Shoreview, MN) and Insight 4GTM software (TSI, Inc., Shoreview, MN), excited the beads using a frame straddling technique. Flow was visualized with a Plan Fluor 20x objective (Nikon, Mellville, NY) coupled to a Powerview 4MP-HS CCD camera (TSI Inc., Shoreview, MN). Due to limitations in the minimum resolvable frame straddling time (“dT”), the maximum flow conditions for each flow type could not be captured, as velocities in these cases exceeded 1 m/s. For shear flow, velocity fields for flow conditions from 0.05 to 0.7 mL/min were captured; for extensional flow, velocity fields were captured for 100 and 200 µL/min. These ranges capture all but four of the experimental flow conditions used for the *in vitro* data collection for this study (Table 2). For each flow condition, 100 image pairs were captured. Images were masked manually by tracing along the inner wall of the flow channel in Insight 4GTM software (TSI, Inc., Shoreview, MN). All processing was completed in Insight 4GTM software: image pairs were cross-correlated using a FFT correlator and processed with a Gaussian peak. Vector conditioning and local validation were also applied during processing.

### In Silico Experiments

**Fig. 3:**
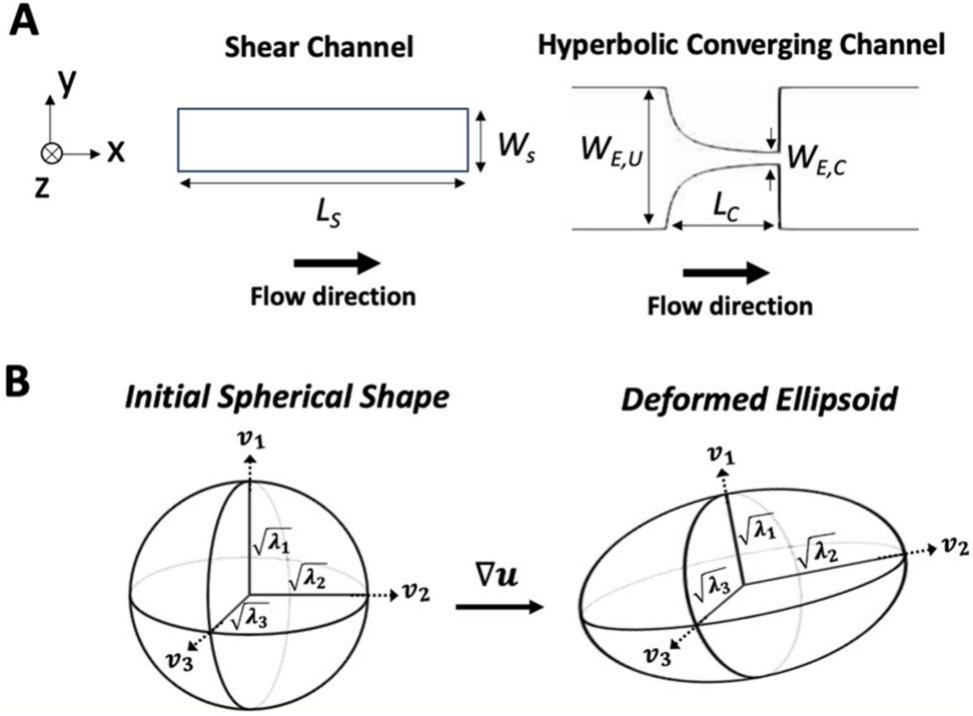
Channel geometries used for *in silico* experiments and visual representation of deformed droplets**. A** Shear and hyperbolic converging microchannels used in flow-induced droplet deformation studies. Channel dimensions: *L*_*S*_ = 1.25 cm, *W*_*S*_ = 400 μm, *W*_*E,U*_ = 2000 μm, *W*_*E,C*_ = 150 μm, and *L*_*C*_ = 1600 μm. Shear and hyperbolic converging channel heights (*h*_*S*_ and *h*_*E*_, into the page*, z*-direction): 50 μm and 35 μm, respectively. The flow direction is shown with a thick black arrow. **B** Representation of the droplet initial state (spherical) and deformed state (ellipsoidal). The square roots of the eigenvalues ($${\lambda }_{i})$$ of ***S*** represent the semi-axes of the deformed ellipsoids. The eigenvectors ($${v}_{i})$$ corresponding to each eigenvalue represent the orientation of the droplets

***Flow Domain Geometries, Governing Equations, and Constitutive Parameters:*** The *in silico* microfluidic channel geometries match those used *in vitro* (Fig. 1B, Fig. 3A). The laminar, incompressible Navier–Stokes equations were solved numerically for steady flow through the channels using the merged pressure-implicit with splitting of operators and semi-implicit method for pressure linked equations (“PIMPLE” method) in OpenFOAM v7.

Maffettone and Minale’s droplet deformation evolution equation (Eq. 3) was coupled to the steady-state flow solutions in the channels and solved in an Eulerian frame [17]. The model assumes that droplets are neutrally buoyant, volume preserving, and deform from a sphere to a triaxial ellipsoid under flow (Fig. 3B). In Eq. 3, ***S*** is a symmetric, 2^nd^ rank tensor whose eigenvalues and eigenvectors represent the ellipsoids’ principal axes’ lengths and orientations, respectively (Fig. 3B). The model is coupled to flow through the fluid velocity (*u*), and the symmetric and antisymmetric parts of the velocity gradient tensor (Eq. 4 and Eq. 5, respectively). The signs on the rotation terms on the left-hand side of Eq. 3 are opposite of those in Maffettone and Minale’s study [17]. This sign change is implemented to remain consistent with the tensorial structure of the velocity gradient in OpenFOAM: Maffettone and Minale define ${(\nabla u)}_{i,j}=\frac{\partial {u}_{i}}{\partial {x}_{j}}$, whereas OpenFOAM source code uses its transpose. As ${{\boldsymbol{W}}}^{T}=-{\boldsymbol{W}}$ and ${{\boldsymbol{E}}}^{T}={\boldsymbol{E}}$, the sign change is made on the rotational terms, while the pure strain terms remain unchanged. A code verification study was conducted to ensure accurate numerical implementation of the droplet deformation model and is detailed in the Supplementary Information Section S.1.

3 $$\frac{{\partial {\boldsymbol{S}}}}{\partial t} + \left( {{\boldsymbol{u}} \cdot \nabla } \right){\boldsymbol{S}} + {\boldsymbol{W}} \cdot {\boldsymbol{S}} - {\boldsymbol{S}} \cdot {\boldsymbol{W}} = - \frac{{f_{1} }}{\tau }\left[ {{\boldsymbol{S}} - g\left( {\boldsymbol{S}} \right){\boldsymbol{I}}} \right] + f_{2} \left( {{\boldsymbol{E}} \cdot {\boldsymbol{S}} + {\boldsymbol{S}} \cdot {\boldsymbol{E}}} \right)$$

4 $${\boldsymbol{E}} = \frac{1}{2}\left( {\nabla {\boldsymbol{u}} + \nabla {\boldsymbol{u}}^{T} } \right)$$

5 $${\boldsymbol{W}} = \frac{1}{2}\left( {\nabla {\boldsymbol{u}} - \nabla {\boldsymbol{u}}^{T} } \right)$$

Deformation is governed via constitutive parameters $${f}_{1}$$ (Eq. 6) and $${f}_{2}$$ (Eq. 7), which scale the sink and source terms of the evolution equation, respectively. The parameters $${f}_{1}$$ and $${f}_{2}$$ are non-negative, unitless values which are required to fully specify the model and are the mathematical result of a first-order expansion of Eq. 3 which can be found in [31]. A dimensionless viscosity ratio is represented by $$\alpha$$ (Eq. 8): the ratio between the viscosity inside the droplet ($${\mu }_{Internal}$$, 6 cP for the RBC cytosol) and the suspending fluid ($${\mu }_{External}$$) [1]. $$\tau$$ is the droplet relaxation time (Eq. 9) and is a relationship between the suspending fluid viscosity ($${\mu }_{External}$$), the droplet interfacial tension ($$\sigma$$), and the droplet initial radius (*R*). The relationship between viscous deforming forces of the external fluid and interfacial restoring force of the droplet and is described with the dimensionless capillary number, *Ca* (Eq. 10). The capillary number (*Ca*) is quantified by multiplying the droplet relaxation time by the Frobenius norm (the magnitude) of the velocity gradient tensor (Eq. 10). Volume preservation of *S* is mathematically enforced with the term *g(S*) (Eq. 11) and is a relationship between its second ($$I{I}_{s})$$ and third ($$I{II}_{s})$$ invariants.

Benchmark simulations were completed using Maffettone and Minale’s evolution equation to evaluate the constitutive parameters’ effect on deformation. Results were compared with *in vitro* RBC deformation data and demonstrated that the original expressions for *f*_*1*_ and *f*_*2*_ required modifications [17]. The form of *f*_*2*_ used in this study (Eq. 7) mirrors the original expression but is scaled with different constants, as results showed that the original asymptotic limits of *f*_*2*_ incorrectly predicted deformation (see Supplementary Information Section S.2). A factor of $\alpha$ was also introduced to the denominator of the second term in the expression for *f*_*2*_ to capture variation across multiple viscosity conditions in high *Ca* flows. The value of *f*_*1*_ remains unchanged at low *Ca*, with modifications implemented as the tensor increases in magnitude to mimic the effect of the cell membrane limiting deformation. A hyperbolic tangent function captured RBC behavior while satisfying the requirement that *f*_*1*_ remain bounded. To further preserve numerical stability at the highest *Ca* in this study, if the maximum eigenvalue of ***S*** (${\lambda }_{L}$) exceeded a critical, nonphysical value (i.e., 250), an artificial relaxation was introduced by setting ${f}_{1}$=1000 (Eq. 6). Importantly, with these modifications implemented, droplet deformation remains physical according to the requirements laid out by Maffettone and Minale: deformation varies linearly with *Ca* in low *Ca* flows and droplet volume is preserved at any *Ca* [17]. Further information on the modifications made to *f*_*1*_ and *f*_*2*_ is available in the Supplementary Information (Section S.2).

6 $$f_{1} = \left\{ {\begin{array}{*{20}c} {50*\tanh \left( {\lambda_{L} *0.8 - 5.656} \right) + 50 + \frac{{40\left( {\alpha + 1} \right)}}{{\left( {2\alpha + 3} \right)\left( {19\alpha + 16} \right)}}, \lambda_{L} < 250} \\ {1000, \lambda_{L} \ge 250} \\ \end{array} } \right.$$

7 $$f_{2} = \frac{4.6\alpha - 0.6}{{\alpha \left( {2\alpha + 3} \right)}} + \frac{{0.25*Ca^{2} }}{{100*\alpha + 1.25*Ca^{2} }}$$

8 $$\alpha = \frac{{\mu_{{{\mathrm{Internal}}}} }}{{\mu_{{{\mathrm{External}}}} }}$$

9 $$\tau = \sigma /(\mu_{{{\mathrm{External}}}} *R)$$

10 $$Ca = \tau *\left| {\nabla {\boldsymbol{u}}} \right|$$

11 $$g\left( {\boldsymbol{S}} \right) = 3\frac{{III_{s} }}{{II_{s} }}$$

***In Silico Experimental Conditions:*** Two viscosity conditions are used in the *in silico* experiments: $\alpha$ = 1.44 and $\alpha$ = 3, which correspond to the conditions used *in vitro,* where the suspending solution viscosities (${\mu }_{External}$) are ~ 4.17 and ~ 2.05 cP, respectively*.* The *in silico* parameters (external fluid viscosity, density, and droplet relaxation times) used for the two viscosity ratio conditions are shown in Table 3. For consistency, the two viscosity conditions will be referred to by their viscosity ratio value ($\alpha =1.44\text{ or }\alpha =3)$ for the duration of this paper. To calculate the droplet relaxation time for each viscosity ratio condition, a physical value for the interfacial tension of the RBC membrane in an aqueous solution is required. There is a wide range of experimentally determined values for the RBC interfacial tension in an aqueous environment, spanning from 3.47 × 10^-6^ to 4.715 × 10^-3^ N/m [32–36]. Using this range, acceptable droplet relaxation times range from 3.54 × 10^-6^ to 1.6 × 10^-2^ s for $\alpha =1.44$ and from 1.70 × 10^-6^ to 8 × 10^-3^ s for $\alpha =3$. Benchmark simulations showed the optimal relaxation times were to be 8.34 × 10^-4^ s and 4 × 10^-4^ s for $\alpha =1.44$ and $\alpha =3$, respectively, and were used in all simulations.

**Table 3:** Fluid viscosity, density, and droplet relaxation times used for the *in silico* experiments

Viscosity ratio ($\alpha )$	Fluid viscosity, ${\mu }_{External}$ (cP)	Fluid density (g/mL)	Droplet relaxation time (×10^-4^ s)
1.44	4.17	1.01	8.34
3	2.0	1.01	4.0

For clarity, the specified relaxation time used in this model represents the tendency of the droplet:fluid interface to minimize its surface area, not the time over which a deformed RBC relaxes back to its resting biconcave shape [17]. This relaxation time is governed by interfacial mechanics and is not an elastic membrane response. The physical difference between this model’s droplet relaxation time and an RBC shape recovery time explains the contrast between our relaxation times and those reported in literature for the RBC membrane, typically in the range of $o\left( {10^{ - 2} } \right) - o\left( {10^{ - 3} } \right)$ s [37]. The implications of using a membrane-free representation to model RBC deformation will be discussed later.

***Discretization Schemes, Boundary Conditions, and Simulations:*** The Navier–Stokes equations were discretized with second-order upwind-based divergence and second-order gradient schemes. The fluid mechanics simulations were solved numerically at steady state with a constant inlet velocity and no-slip condition at the walls. The pressure at the inlet was zero and at the outlet a homogeneous Neumann boundary condition was used with a gradient of zero (“zero gradient” in OpenFOAM). For the deformation simulations, the initial condition and the inlet boundary condition of ***S*** was set to the identity tensor, representing a sphere. A homogeneous Neumann boundary condition of zero gradient was used at channel walls and outlet boundary for ***S*** (“zeroGradient” boundary condition in OpenFOAM). The deformation simulation equations were discretized with second-order upwind-based divergence schemes and second-order gradient schemes. All simulations were run for at least 20 characteristic relaxation times ($$\tau$$), and the shape tensor (*S*) data were then sampled in the geometries at defined locations. All deformation is quantified using a deformation index (DI), based on the relationship between the semi-axes of the deformed ellipsoids (the square root of the eigenvalues of the morphology tensor $$\sqrt{{\lambda }_{i}}$$; Fig. 3B$$)$$. Twenty relaxation times were used as a cutoff as it allowed the solution to develop to a steady state. A computational mesh refinement study was performed to ensure the numerical solutions were independent of the meshes used in both channel types and is detailed in the Supplementary Information (Section S3). Simulations were run on OpenFOAM v7 on two platforms for this study: Penn State’s Institute for Computational and Data Sciences Roar Collab and Texas Advanced Computing Center Stampede3 systems. Simulations were run for each flow rate in the shear and extensional flow conditions (Table 1) for both viscosity conditions (28 simulations total). The *in silico* data from these 22 shear simulations and 6 extensional flow simulations were then compared to the *in vitro* data collected in this study at the same conditions.

In order to reach higher extensional strain rates for MCSD relevance, additional simulations were run for $\alpha =1.44$ at four flow rates: 1.26, 2.1, 2.6, and 4.2 mL/min, corresponding to 2*${\dot{\gamma }}_{Extension}$ = ~ 4,040, 6,695, 8,360, and 13,160 s^-1^, respectively. Due to high RBC velocities, our *in vitro* imaging microscopy system could not capture cell images at flow rates in extension above 400 µL/min without significant blur. Therefore, simulation data in these flows were compared to published *in vitro* RBC deformation data that were collected in a hyperbolic converging microchannel for $\alpha =1.44$ only at strains from ~ 900 to 10,000 s^-1^ [14].

***Model Accuracy Metrics:*** All *in vitro* data in this study are reported as mean and the corresponding standard error of the mean of the data set (SEM, Eq. 12). In Eq. 12, *SD* is the standard deviation of the sample data set and *n* is the number of experimental replicates (i.e., *n* = 6 experiments). To analyze the accuracy of the *in silico* droplet deformation results, an assessment was performed to determine whether the *in silico* data were within the range specified by the mean ± SEM in that condition.

12 $$SEM = \frac{SD}{{\sqrt n }}$$

Further, to understand the average difference between the *in vitro* DI values and the *in silico* prediction, the mean absolute error (MAE, Eq. 13) was calculated. To calculate MAE, the difference between the *in silico* DI value (${\widehat{DI}}_{In Silico}$) and each individual RBC DI measurement (${{DI}_{In Vitro}}_{i}$) is summed for all RBCs in each condition. This value is then divided by the number of RBCs analyzed (${n}_{RBCs}$=300). The MAE was calculated for the DI data in Planes 1 and 2 for both *α* = 3 and *α* = 1.44 across all shear and extensional flow conditions and imaging locations.

13 $$MAE = \frac{1}{{n_{RBCs} }}\mathop \sum \limits_{i = 1}^{{n_{RBCs} }} \left| {DI_{{In\,\,\,Vitro_{i} }} - \hat{D}I_{In\,Silico} } \right|$$

## Results

### Comparison of In Silico and In Vitro Velocity Data

**Fig. 4:**
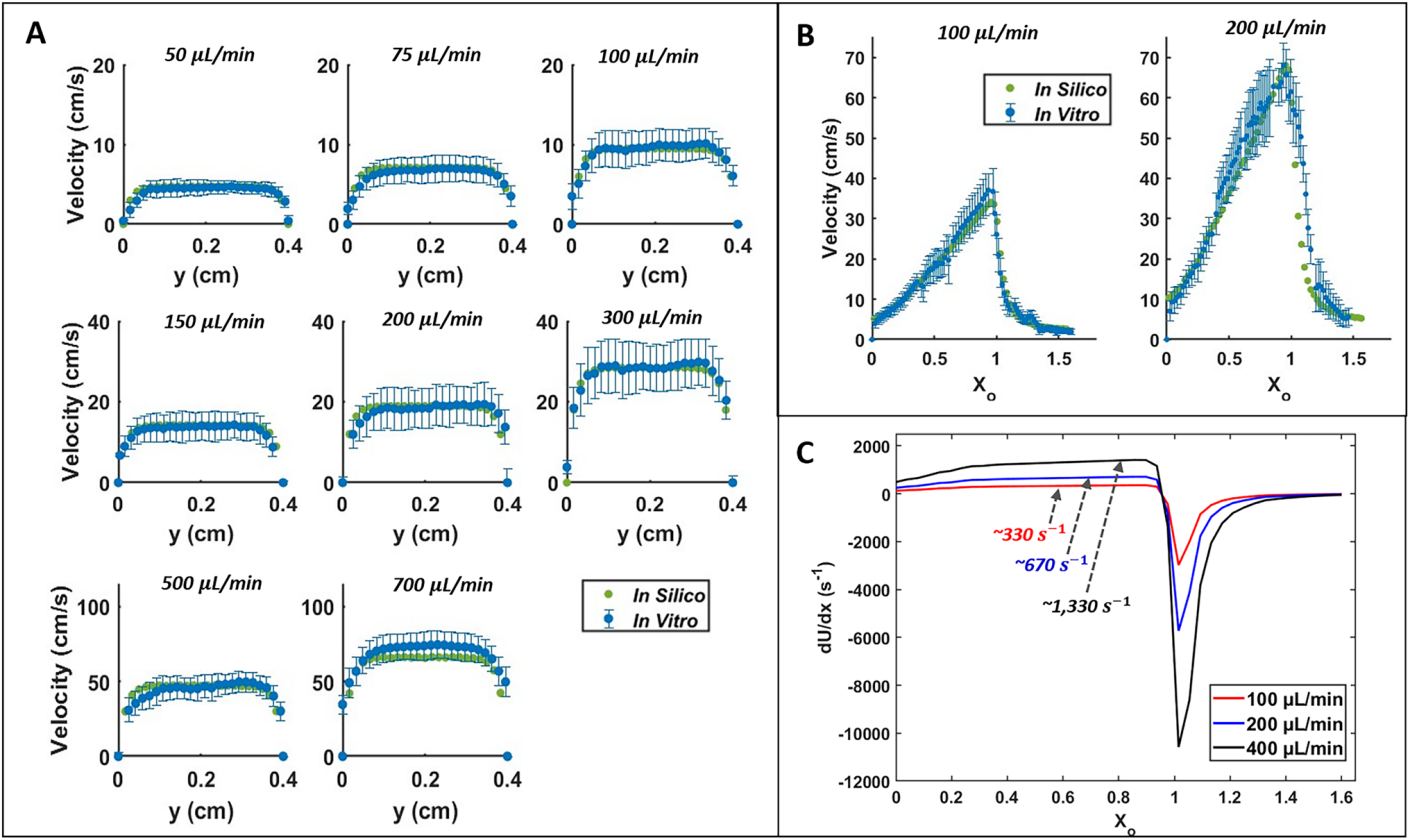
Comparison of *in silico* and *in vitro* velocity data, along with *in silico* velocity gradient data along the centerline of the hyperbolic converging microchannel. **A** *In vitro* and *in silico* averaged velocities across the shear channel width (*W*_***s***_) for 8 flow conditions. **B** *In vitro* and *in silico* average velocities along the length of the hyperbolic converging microchannel (*L*_*c*_) for 2 flow conditions. **C** *In silico* results for the first component of the velocity gradient tensor $(\frac{\partial U}{\partial x}$) along the centerline of the hyperbolic converging channel in each flow condition. The plateau value of the velocity gradient from X_o_ = 0.5–1 is notated using a dashed arrow for each flow rate. (Note: the axis values are altered through the figure for readability. The error bar is the standard deviation of the velocity measurements in panel A and B of this figure).

Figure 4A compares the *in vitro* and *in silico* velocity profiles across the channel width for shear flow. The data presented are the average velocity across the width of the channel (*W*_*s*_), and the *in vitro* data are presented with an error bar corresponding to the standard deviation of the velocity measurements. Figure 4B shows the averaged velocity in the hyperbolic converging channels along the channel’s contraction length (*L*_*c*_) for both *in silico* and *in vitro* data. Excellent agreement is found between *in silico* and *in vitro* velocity data, with the *in silico* velocity data falling within the standard deviation of the *in vitro* data in each case.

### Hyperbolic Converging Microchannel Velocity Gradients

An analysis of the fluid strain rate along the centerline of the extensional flow channel using the *in silico* results is shown in Fig. 4C. The only non-zero components of the velocity gradient tensor along the centerline of the channel are $\frac{\partial U}{\partial x}$ and $\frac{\partial V}{\partial y}$, where $\frac{\partial U}{\partial x}$= -$\frac{\partial V}{\partial y}$= 2*${\dot{\gamma }}_{Extension}$ for 0.5 < X_o_ < 1 (Table 2). The velocity gradient ($\frac{\partial U}{\partial x})$ plateaus at ~ X_o_ = 0.5 in each condition, matching the analytical values in Table 2 (Fig. 4C). The flow then abruptly decelerates when it reaches the sudden expansion, reflected through a highly negative $\frac{\partial U}{\partial x}$ when X_o_ > 1.

### Comparison of Deformation Data Between In Silico Droplets and Human RBCs in Shear Flow

**Fig. 5:**
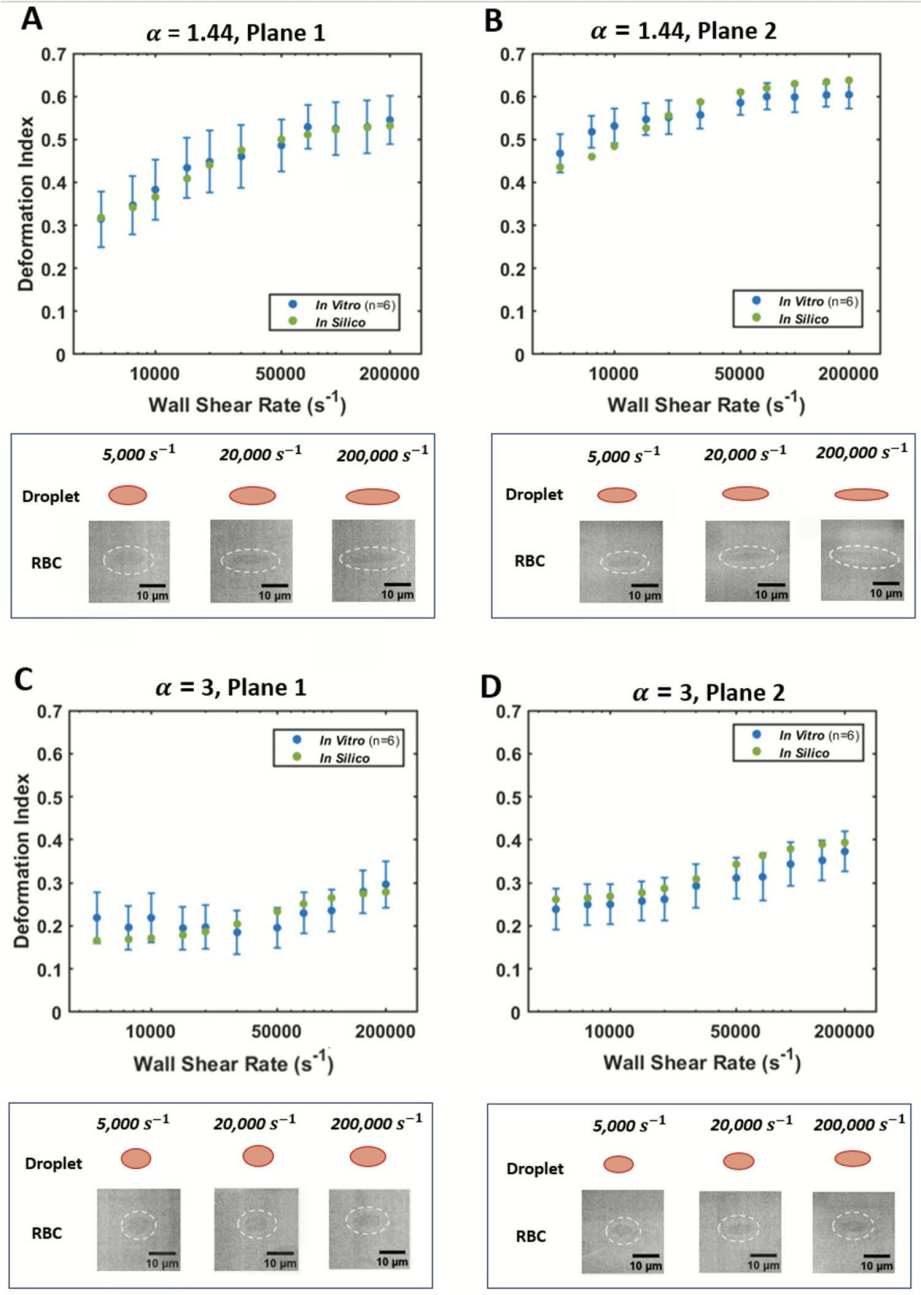
Comparison of *in silico* droplet and *in vitro* RBC deformation (*n* = 6) in shear flow from 5,000 to 200,000 s^-1^. Each *in vitro* data point represents the average DI quantified from 300 RBCs at the specified flow condition. The error bar represents the SEM for the 300 RBCs processed for each flow condition. Example RBC images (*in vitro)* and deformed droplets (*in silico*) are shown for each viscosity condition in both planes below the plots at 5,000 s^-1^, 20,000 s^-1^, and 200,000 s^-1^ for a visual representation of the deformation. A dashed white oval is traced around each *in vitro* RBC. **A, B** Deformation data for $\alpha =1.44$ in Planes 1 and 2, respectively. **C, D** Deformation data for $\alpha =3$ in Planes 1 and 2, respectively. (Note: the *x*-axis uses a log scale)

*In silico* droplet and *in vitro* RBC deformation in shear flow are shown in Fig. 5 for both viscosity ratios ($\alpha$=1.44, 3) and visualization planes (Plane 1, Plane 2). Example RBC images and deformed droplets are shown in each panel of Fig. 5 for 5,000, 20,000, and 200,000 s^-1^ to visualize the progressive deformation in either viscosity and/or plane. Each RBC image is outlined with a dashed white oval. Each *in vitro* data point represents the average DI quantified from 300 RBCs at the specified flow condition. For these experiments, the wall shear stress (WSS) in the channel ranges from 210 to 8,340 dyne/cm^2^ for $\alpha$=1.44 and from 100 to 4,000 dyne/cm^2^ for $\alpha$=3. Due to the high velocity gradients, the cells’ orientation angle in all cases was less than 5° with respect to the flow direction at all conditions, which agrees with the orientation result produced by the droplet deformation model (data not shown).

*In silico* deformation data agree with the average *in vitro* DI values, falling within the measurement mean ± SEM for all flow conditions in Plane 1 and Plane 2, except at 7,500 s^-1^ in Plane 2 for $\alpha$=1.44, where there is an 11.3% difference in *in vitro* and *in silico* DI. The MAE values in both Plane 1 and Plane 2 for both α = 1.44 and *α* = 3 across the 11 shear flow conditions are detailed in Supplementary Information Section S.4. The MAE range across the 11 shear flow conditions in both Plane 1 and Plane 2 spans from 0.05 to 0.15 for *α* = 1.44 and from 0.08 to 0.11 for *α* = 3. The MAE magnitudes did not show a specific trend across the flow conditions in either Plane 1 or Plane 2 for *α* = 1.44 and *α* = 3.

The MAE magnitude in relation to the average DI value is attributed to high RBC variability with standard deviations up to 50% of the average DI values across the shear flow conditions. Inter-donor variability in RBC DI was analyzed using intraclass correlation coefficients and demonstrated that 1–34% of the variability in shear flow DI was attributable to inter-donor differences, with the remainder of the variability attributed to intra-donor blood cell variance (Supplementary Information Section S.5).

Deformation is higher across all flow conditions for $\alpha$=1.44 compared with $\alpha$=3, owing to the higher solution viscosity. The cell length in the streamwise direction increases from ~ 10 to ~ 20 μm in the highest shear conditions for $\alpha$=1.44 (Fig. 5A, B). In contrast, the lengthwise cell dimension increases only a few microns for $\alpha$=3 (Fig. 5C, D). The cell deformation tends to reach a threshold at around DI ~ 0.60 for $\alpha$=1.44 in Plane 2 that does not increase significantly between 70,000 and 200,000 s^-1^ (Fig. 5A, B). The DI is slightly higher in Plane 2 for both viscosity ratios.

### Extensional Flow-Induced RBC Deformation: Comparison Between In Silico Results and In Vitro Measurements

**Fig. 6:**
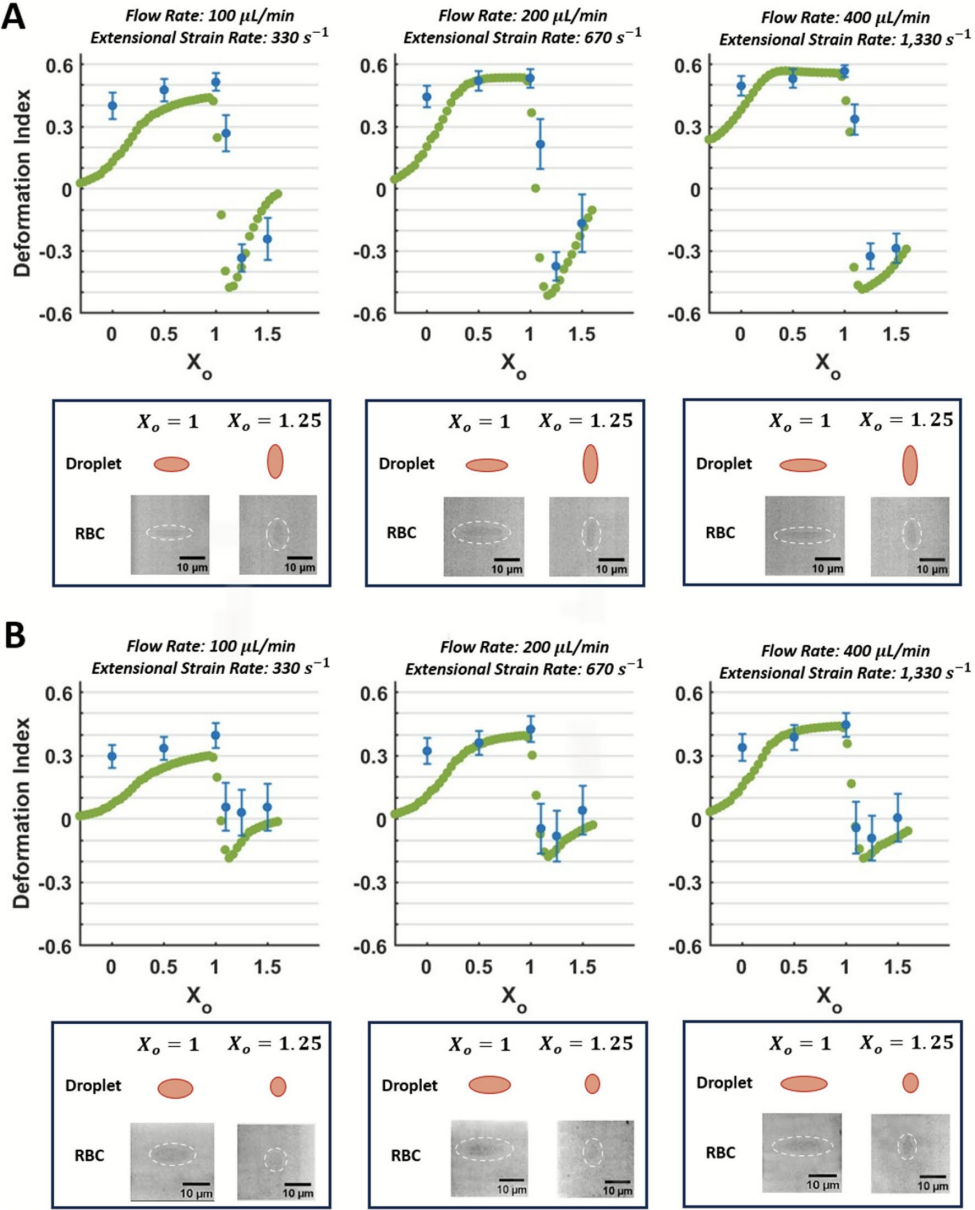
Comparison of *in silico* droplet deformation and *in vitro* RBC deformation at $\alpha$ = 1.44 in extensional flow. Deformation data are plotted along the length of the hyperbolic converging microchannel using a normalized axial positioning system (X_o_). Each *in vitro* data point represents the average DI quantified from 300 RBCs at that location and flow rate. Examples of deformed RBCs and droplets are shown for X_o_ = 1 and X_o_ = 1.25 in both planes, and the *in vitro* RBCs are outlined with a dashed white oval. The error bar represents the SEM. **A** Deformation data for $\alpha =1.44$ in Plane 1 across the three flow conditions.** B** Deformation data for $\alpha =1.44$ in Plane 2 across the three flow conditions

*In silico* droplet and *in vitro* RBC deformation at each extensional flow condition and in both planes are shown in Fig. 6 for $\alpha =1.44$. The plateaued extensional strain rate in the throat of the channel (as shown in Table 2, Fig. 4C) is also notated. Data are plotted along the hyperbolic converging channel’s contraction length using the normalized axial positioning system (X_o_), where X_o_ < 1 signifies the region upstream of the sudden expansion and X_o_ > 1 is downstream (Fig. 1C). Example images of stretched RBCs and droplets at the throat of the contraction (X_o_ = 1) and when they are compressed (X_o_ = 1.25) are shown for each condition, and each *in vitro* RBC is outlined with a white-dashed oval. Each *in vitro* data point represents the average DI quantified from 300 RBCs at that location and flow rate.

Across the three flow conditions, cell deformation increases incrementally for the locations measured between 0 < X_o_ < 1, with peak extension (at X_o_ = 1) ranging between 0.51 and 0.58 in Plane 1 (Fig. 6A). The increase in deformation with increasing flow between 0 < X_o_ < 1 is reflected in the *in silico* data for both planes and can be observed in the example RBC images. The deformation behavior again differs between Planes 1 and 2. Plane 1 shows a higher deformation magnitude as it represents both components of the velocity gradient that induce extensional flow ($\frac{\partial U}{\partial x} \mathrm{and} \frac{\partial V}{\partial y}$), while Plane 2 captures the cells’ extension in the streamwise direction ($\frac{\partial U}{\partial x}$) without the directly proportional change in the *y*-direction. The increased deformation in Plane 1 is also due to the cells’ alignment within the channel: the RBC preferentially aligns its ~ 2 $\upmu$ m biconcave thickness parallel to the width of the channel (*W*_*E,C*_ and *W*_*E,U*_).

*In silico* droplet deformation in the hyperbolic converging region (X_o_ < 1) is within the SEM of the *in vitro* data for 200 and 400 $\upmu$L/min, except at X_o_ = 0 and at X_o_ = 1.25 at 400 $\upmu$L/min. *In silico* results fall outside of the SEM of *in vitro* data for 100 $\upmu$ /min from 0 $\le$ X_o_ $\le$ 1, with DI at X_o_ = 1 ~ 20–25% higher *in vitro* than *in silico*. At the contraction throat (X_o_ = 1), the MAE in Plane 1 for 100, 200, and 400 $\upmu$L/min is equal to 0.12, 0.09, and 0.06, respectively; the MAE in Plane 2 for 100, 200, and 400 $\upmu$L/min is equal to 0.14, 0.11, and 0.11, respectively.

The cells experience a dramatic decrease in deformation and subsequent compression after exiting the throat and entering the sudden expansion region (X_o_ > 1). The cells immediately begin to relax at the cessation of extensional, stretching flow around X_o_ = 1, reflected through a decreasing DI, and then compress near X_o_ = 1.1–1.25. The behavior is due to the local flow environment: the velocity gradient tensor has only two non-zero components along the channel’s centerline ($\frac{\partial U}{\partial x}=-\frac{\partial V}{\partial y},$ where $\frac{\partial V}{\partial y}$< 0 for X_o_ < 1), and the signs of the components switch ($\frac{\partial U}{\partial x}<0,\frac{\partial V}{\partial y}>0$) when the flow starts to decelerate (X_o_ = 1), resulting in cell compression (Fig. 4C, Fig. 6). Real-time imaging experiments were conducted to confirm the negative DI in this region was caused only by RBC compression and not by RBC rotation, and video data of the compressing cells along the channel centerline at X_o_ > 0 are available in the Supplementary Information (Video S1). *In silico* droplets also did not rotate in this region, as there is no rotational component of the flow along the channel’s centerline. The cells’ relaxation at X_o_ = 1.1, followed by compression at X_o_ = 1.25, and recovery leading up to X_o_ = 1.5 are reflected in the *in silico* results.

When RBCs are recovering (X_o_ = 1.5), the MAE in Plane 1 for 100, 200, and 400 $\upmu$L/min is equal to 0.19, 0.15, and 0.08, respectively; and the MAE in Plane 2 for 100, 200, and 400 $\upmu$L/min is equal to 0.17, 0.16, and 0.08, respectively. The detailed MAE magnitudes are again supported by high RBC variability: the standard deviations of the RBC measurements ranged from 13 to 20% and 20 – 200% of the mean DI value across the flow conditions at X_o_ = 1 and X_o_ = 1.5, respectively. Inter-donor RBC deformability variability accounted for 10 – 28% of the DI variability at X_o_ = 1 and 8 – 33% of the DI variability at X_o_ = 1.5 (Supplementary Information Section S.5).

Regardless of the flow condition, RBCs in the compressive area of the flow all have DI > − 0.45, although the compressive fluid strain rate varies by an order of magnitude between the three flow conditions (Fig. 4C, Fig. 6). This deformation magnitude is reflected in the *in silico* results, where DI > − 0.5.

**Fig. 7:**
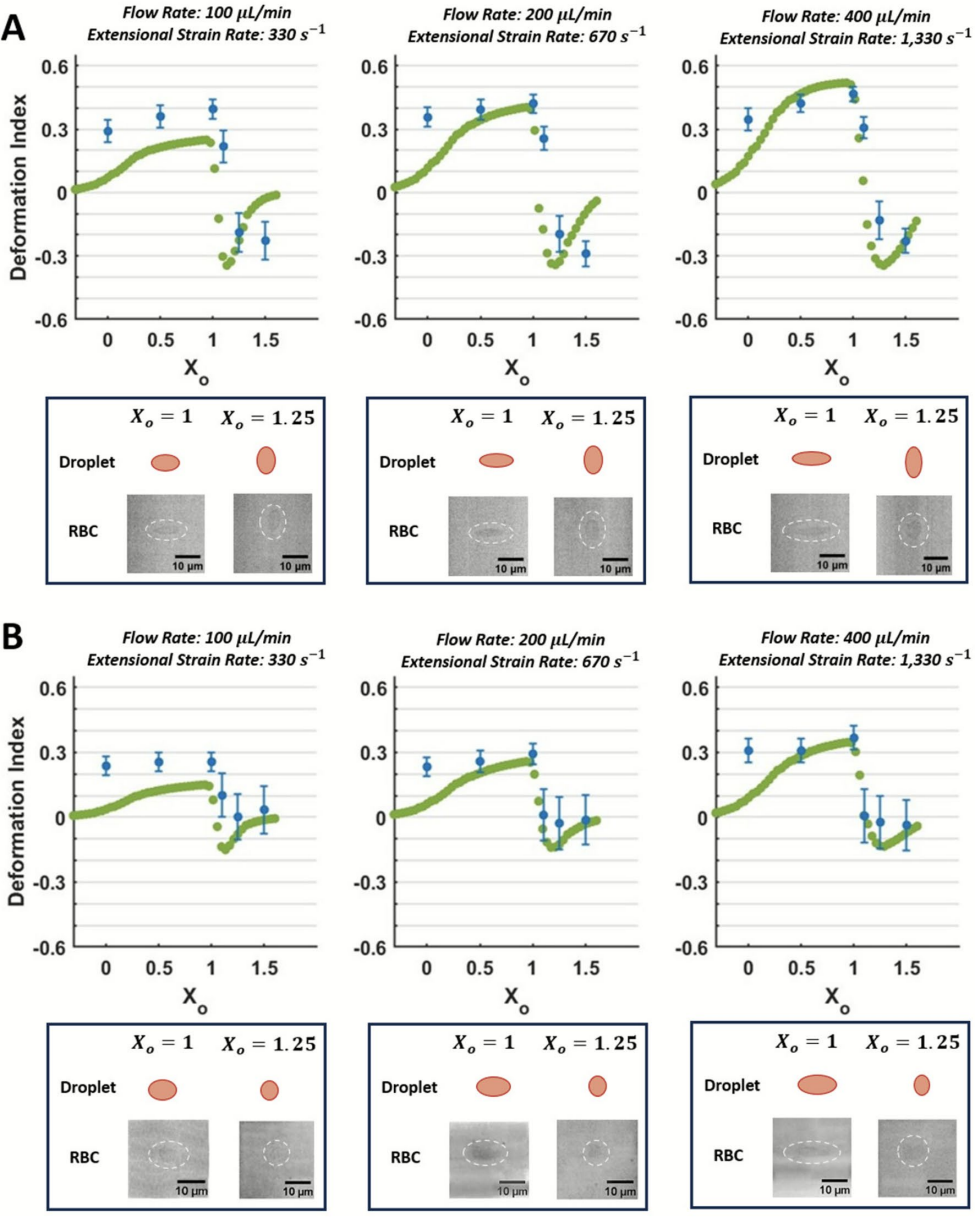
Comparison of *in silico* droplet and *in vitro* RBC deformation at $\alpha =$ 3 in extensional flow. Deformation data are plotted along the length of the hyperbolic converging microchannel using a normalized axial positioning system (X_o_). Each *in vitro* data point represents the average DI quantified from 300 RBCs at that location and flow rate. Example Examples of deformed RBCs and droplets are shown at X_o_ = 1 and X_o_ = 1.25 in both planes, and the *in vitro* RBCs are outlined with a dashed white oval. The error bar represents the SEM. **A** Deformation data for $\alpha =3$ in Plane 1 across the three flow conditions.** B** Deformation data for $\alpha =3$ in Plane 2 across the three flow conditions

*In silico* droplet deformation with *in vitro* RBC deformation in extensional flows in both planes for $\alpha =3$ are shown in Fig. 7. Example images of deformed RBCs and droplets in both planes are again shown for X_o_ = 1 and X_o_ = 1.25. Each *in vitro* data point represents the average DI quantified from 300 RBCs at that location and flow rate. The DI magnitude throughout the entire hyperbolic converging microchannel in both planes across all three flow conditions is lower for $\alpha =3$ as compared to $\alpha =1.44$ (Figs. 6 and 7). Despite the differences in viscosity conditions, trends among the two conditions remain similar: *in vitro* and *in silico* deformation data increase incrementally across the three flow conditions, and deformation in Plane 2 (Fig. 7B) is lower than that in Plane 1 (Fig. 7A). *In silico* data at 100 µL/min are not within the SEM of *in vitro* data between 0 < X_o_ < 1 (Figs. 6, 7). However, for 200 µL/min and 400 µL/min, the numerical results at X_o_ = 0.5 and X_o_ = 1 are within the SEM of the *in vitro* data (Fig. 7). At the contraction throat (X_o_), the MAE in Plane 1 for 100, 200, and 400 $\upmu$L/min is equal to 0.17, 0.09, and 0.07, respectively; the MAE in Plane 2 for 100, 200, and 400 $\upmu$L/min is equal to 0.13, 0.08, and 0.1, respectively. The compression and recovery of the cells (X_o_ > 1) for $\alpha =3$ is different to those in $\alpha =1.44$, owing to the viscosity of the solutions affecting the compressive behavior of the cell dynamics in these regions (Figs. 6 and 7). The compression (DI < 0) and cell recovery (recovering from DI < 0 to DI = 0) occur over a longer spatial distance in this viscosity condition. The *in silico* data in the downstream channel region (X_o_ > 1) match the *in vitro* data at the highest flow condition (400 $\upmu$L/min). The MAE at X_o_ = 1.5 in Plane 1 for 100, 200, and 400 $\upmu$L/min is equal to 0.18, 0.13, and 0.05, respectively, and the MAE at X_o_ = 1.5 in Plane 2 for 100, 200, and 400 $\upmu$L/min is equal to 0.18, 0.15, and 0.08, respectively.

The standard deviations of the RBC measurements ranged from 31 to 47% and 20 to 180% of the mean DI value across the flow conditions at X_o_ = 1 and X_o_ = 1.5, respectively. Inter-donor RBC deformability variability accounted for 4 – 15% of the DI variability at X_o_ = 1 and 6 – 35% of the DI variability at X_o_ = 1.5 (Supplementary Information Section S.5).

**Fig. 8:**
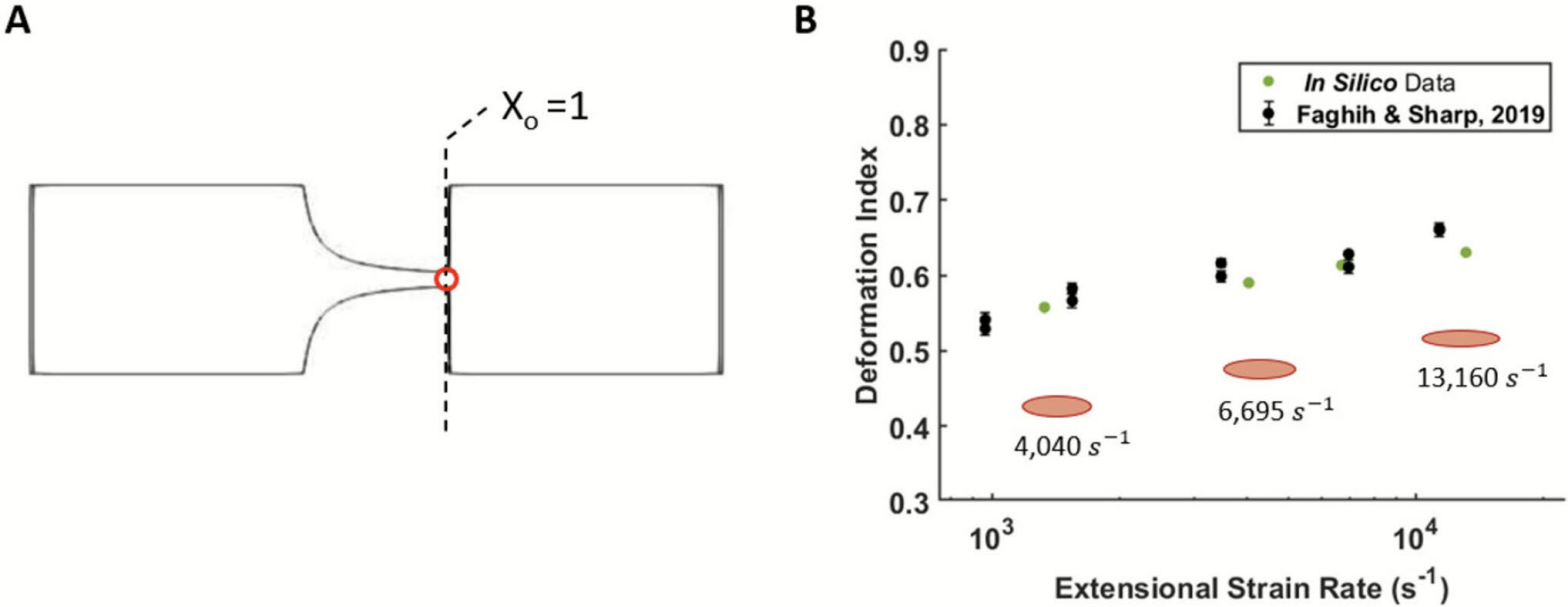
Comparison of numerical and *in vitro* deformation data in extensional flows at strain rates up to 13,160 s^-1^ for $\alpha =1.44$. Experimental data are from Faghih and Sharp [14].** A** Visual representation of the sampling location at X_o_ = 1 (red circle) for the images taken in the *in vitro* study [14] and for the numerical data presented. **B** Numerical and *in vitro* data from ~ 950 to ~ 13,000 s^-1^ extensional strain rate. *In vitro* data were from two human blood samples at each strain rate. The error bar is standard deviation from the published *in vitro* study. A representative deformed droplet is shown for 4,040, 6,695 and 13,160 s^-1^ with a red ellipse. (Note: the *x*-axis uses a log scale)

To capture deformation at higher extensional strain rates relevant to MCSDs, additional simulations were performed for $\alpha =1.44$ and compared to published *in vitro* data [14] at the same conditions; the data are shown in Fig. 8. Deformation data in the *in vitro* study were collected at the throat of a hyperbolic converging microchannel (X_o_ = 1) (Fig. 8A). The suspensions used in the *in vitro* study were also from human blood samples and dilute hematocrit (${\mu }_{External}$ = 4.16 cP) [14]. Experimental DI reaches ~ 0.65 and the numerical data agree well at these strain rates, reaching ~ 0.61. There are not MAE values to quantify here as only the average DI data are presented in this experimental study; however, the *in silico* DI values are within 7% of the average *in vitro* DI across these flow conditions (Fig. 8B). An example deformed droplet is shown in Fig. 8B for 4,040, 6,695, and 13,160 s^-1^.

## Discussion

In this study, the utility of Maffettone and Minale’s droplet deformation model for predicting *in vitro* RBC deformation is presented. Modifications were made to the constitutive model parameters to best capture RBC deformation in both shear and extensional flows for two viscosity conditions. Advanced high-speed imaging and post-processing techniques were used to build a set of high-fidelity three-dimensional RBC deformation data for model calibration across a suite of conditions. Supraphysiological strain rates were primarily investigated in this study with the intent to broaden the approach to MCSD flow-induced RBC damage and hemolysis modeling.

### Deformation of Human RBCs Compared to In Silico Droplets in Shear Flows

Comparing *in silico* and *in vitro* data in canonical flows is important for accurately accessing this model’s ability to represent human RBC deformation behavior. First, *in silico* data in shear flow are in excellent agreement with *in vitro* data, with only 1 of 44 total shear data points falling outside of the *in vitro* SEM ($\alpha$=1.44 in Plane 2 at 7,500 s^-1^, Fig. 5). The MAE values across the shear flow experiments reach 0.15 for $\alpha$=1.44 and 0.11 for $\alpha$=3; however, these metrics result from inherently high RBC DI variability, and *in silico* DI still remains within the spread of the data (mean ± SEM) in almost all cases. Additionally, a DI magnitude equal to 0.15 or 0.11 represents a nearly circular, slightly deformed cross section, demonstrating that our model on average predicts an ellipse that is only slightly different to the deformation of the *in vitro* RBCs.

For $\alpha$=1.44, both the *in vitro* and *in silico* shear deformation data reach a threshold DI of ~ 0.54 in Plane 1 and ~ 0.60 in Plane 2 at 70,000 s^-1^. These DI magnitudes do not continue to increase, even when the shear rate is 200,000 s^-1^ (Fig. 5A, B). These data agree with published *in vitro* deformation thresholds, where RBC DI does not exceed ~ 0.60 in similar viscosity and strain rate conditions [14, 27]. The phenomenon observed here is physically attributed to the RBC’s resistance to areal dilation: the RBC membrane will deform with increasing fluid strain until additional deformation requires an increase in membrane surface area, at which point the RBC membrane’s high areal dilation modulus resists deformation [1, 38]. The numerical model’s parameters have been modified such that these physical deformation thresholds are represented *in silico.* The DI values are lower in $\alpha$=3, due to a decreased viscous friction on the membrane at the same strain rates. Accurate DI for $\alpha$=3 is reproduced in the *in silico* simulations, as the constitutive parameter *f*_*2*_ has a direct dependence on the viscosity of the suspending solution to tune the deformation source. The shear DI data for $\alpha$=3 do not reach a threshold, demonstrating the effect of solution viscosity on deformation behavior. In this viscosity condition, a much higher shear rate may be required to deform the RBCs to the threshold DI value. However, in either viscosity condition, the asymptotic limits of deformation in the numerical model are set such that the droplet DI will not surpass ~ 0.60 in shear flow to match the described physiological RBC phenomenon.

### Deformation of Human RBCs Compared to In Silico Droplets in Extensional Flows

The results in extensional flow first help identify under what strain rates the numerical approach is most accurate. Data show that at peak extension (X_o_ = 1) and upon droplet recovery (X_o_ = 1.5), model MAE generally decreases as the extensional strain rate increases. Specifically, in both Plane 1 and Plane 2 at 100 µL/min (330 s^-1^ extensional strain rate), the MAE in peak extension (X_o_ = 1) ranges from 0.12 to 0.17 for both viscosity conditions, while the range is between 0.06 and 0.11 for 200 and 400 µL/min (670 and 1,330 s^-1^, respectively). Further, upon droplet/RBC recovery (X_o_ = 1.5), the model MAE in both Plane 1 and Plane 2 ranges between 0.17 and 0.19 for 100 µL/min (330 s^-1^ extensional strain rate) for both viscosity conditions, while the range is between 0.05 and 0.16 for 200 and 400 µL/min (670 and 1,330 s^-1^, respectively). This analysis did not show that either of the visualization planes or viscosity conditions had consistently higher MAE, rather, only that MAE generally decreases for peak extension (X_o_ = 1) and recovery (X_o_ = 1.5) as the flow condition increased. The implications of the MAE values in extensional flows are similar to the discussion presented for shear flow: the MAE magnitude represents the wide RBC variability and only a slight shape difference between the *in silico* ellipse and a highly deformed RBC (DI up to ~ 0.6), with the caveat in extensional flows that the MAE decreases when modeling extensional strain rates above 330 s^-1^.

The error at lower extensional strain rates is related likely to the challenges in modeling the resting biconcave disc, which has been found in previous studies [39]. The unique shape of the biconcave disk as compared to a slightly deformed sphere at low strain rates (i.e., 330 s^-1^) produces higher deformation modeling errors, which are reduced in higher strain rate flows when both the cells and droplets are ellipsoidal. Additionally, at 330 s^-1^, the RBC behaves less like a deformable liquid droplet and more like a capsule with a membrane that can resist low magnitude flow-induced deformations, making it more challenging to replicate *in vitro* data using this modeling approach. This has also been shown in previous *in vitro* studies [27].

Further, the data presented demonstrate principal differences in RBC dynamics at differing viscosities. For $\alpha$=1.44, *in vitro* and *in silico* data demonstrate sequential stretching, relaxation, compression, and recovery of the RBCs and droplets through the channel (Fig. 6). Specifically, the DI is positive for 0 < X_o_ < 1, becomes negative between 1.1 < X_o_ < 1.25, and then increases from 1.25 < X_o_ < 1.5. The stretching behavior in the channel throat followed by compression in a sudden expansion has also been identified with human RBCs in the previous work [27]. This behavior occurs in both planes but is more dominant in Plane 1, which visualizes the main extensional/compressive velocity gradients (Fig. 6A, B). In contrast, RBC deformation in $\alpha$=3 takes a longer streamwise distance to reach full compression, and thus, DI data do not significantly increase between 1.25 < X_o_ < 1.5 (Fig. 7). The dominant source of deformation for $\alpha$=1.44 is likely the suspension viscosity, which immediately compresses cells exiting the hyperbolic converging channel’s throat, and then exerts a viscous force aiding in recovery between 1.25 < X_o_ < 1.5. For $\alpha$=3, the viscous friction on RBCs is decreased, and cell compression takes a longer streamwise distance when compared to the same flow conditions in $\alpha$=1.44. However, the numerical deformation data do show recovery of the droplets as the DI reaches an inflection point around X_o_ = 1.25 for $\alpha$=3 for all flow conditions (Fig. 7), which is again likely due to modeling membrane-free liquid droplets.

Although there is an elastic restoring component in the governing equation, there is not an explicit droplet membrane which could resist compression and govern droplet recovery. As a result, the numerical model better replicates the overall *in vitro* behavior through the entire channel in flows where the viscosity of the suspending solution directly deforms the cell membrane ($\alpha$ = 1.44). The model physically represents RBCs with both viscous and elastic components, and constitutive parameters were tuned to represent human RBCs. However, the approach has difficulty in less viscous and/or lower strain rate flows where the membrane can resist deformation more easily. In any case, the errors found at the likely non-hemolytic flows (330 s^-1^ extensional strain rate) were of minor concern in this study, as the main application of this numerical model will be toward MCSD flows where lysis will typically occur at supraphysiological strain rates. For *in silico* studies of medical device flows, it is possible that corrections will need to be made to cell damage or hemolysis predictions based on the percentage of the device’s flow domain that is < 330 s^-1^.

Further, the hyperbolic converging microchannel provides an environment that is replicative of MCSD flows for analysis of RBC deformation: there are spatially varying, high magnitude strain rates, which the RBCs experience for short exposure times (~1–10 ms). In this environment, the extensional DI data also reach a threshold for $\alpha$=1.44. For example, *in vitro* deformation does not significantly increase between 1,000 and 13,000 s^-1^ extensional strain (Fig. 8). Additionally, at the highest magnitude compressive fluid strain (1.1 < X_o_ < 1.25), both the *in silico* and *in vitro* data do not reach DI < − 0.50 for $\alpha$=1.44 (Fig. 6). The *in silico* data are in agreement with these *in vitro* data, demonstrating that the modifications made to the model’s constitutive parameters continue to capture RBC deformation thresholds and DI magnitudes at MCSD relevant strain rates in both extension and compression.

Finally, an important aspect of the numerical approach presented here is the ability to replicate the significantly different RBC deformation behaviors in shear and extensional flows. The *in vitro* data in this study and previous studies demonstrate the different deformation magnitudes in shear and extensional flows using human RBCs [14, 16]. For example, at 200,000 s^-1^ in shear flow for $\alpha$=1.44, the DI in both planes varies from 0.55 to 0.6, while at only 1,330 s^-1^ in extensional flow the DI is approximately equivalent (Figs. 5, 6). The increasing *f*_*1*_ value which scales the deformation sink in Eq. 3, along with the equation which bounds *f*_*2*_ in high *Ca* flows, allows the model to represent the deformation in both shear and extension over multiple orders of magnitude of fluid strain rate.

### Implications of Intra-Donor RBC Variability for Deformation Modeling

The variability of individual RBC measurements in this study was high, reaching up to 200% of the average DI value in some cases. This variability was constant in shear flow and generally increased in the hyperbolic converging microchannel when cells were compressed (SEM bars widen in Figs. 6, 7 for X_o_ ≥ 1.1 vs. X_o_ ≤ 1), suggesting that not all RBCs display the same relaxation and compression dynamics. Further, the distributions in the data show that there is high heterogeneity in RBC populations and that modeling the RBC deformation behavior will have MAE ~ 0.05 – 0.16 due to this variability. As these MAE values are much lower than the standard deviations in many of the data sets and the *in silico* data falls within the SEM of the data points, these errors are deemed acceptable.

The source of the variability is of interest for this study and future work in modeling RBC deformability. Previous work has shown that intra-donor variability (i.e., how much one donor’s cells vary from one another) is higher in magnitude than inter-donor variability for hemolysis testing *in vitro*. Our study follows a similar trend, where intraclass correlation coefficients (see Supplementary Information Section S.5) showed there is a greater influence in variance from intra-donor differences as compared to inter-donor differences. As such, this study suggests that the variability observed in the RBC deformation measurements is likely due to a wide population of cells naturally present in donor blood, rather than large differences between different donors. The findings also suggest that the intra-donor variation and data set spread (standard deviation, SEM), should both be considered in order to model RBC deformability within reasonable error values.

### Comparison to Previous Droplet Deformation Modeling Approaches

Distinctions should be made between the study presented here and published work using Maffettone and Minale’s droplet deformation model in MCSD hemolysis simulations [18–23]. The primary difference between the approach used here and prior work using this droplet deformation model is the constitutive parameters that scale the source and sink terms of Eq. 3. For example, Pauli *et al.* implemented slight modifications to the droplet deformation model, absorbing $\tau$ into the parameter scaling the deformation sink (*f*_*1*_) and adding an additional scaling factor *f*_*3*_ on the rotational terms of the equation [18]. Values for these parameters were as follows: *f*_*1*_ =5 s^-1^; *f*_*2*_ = *f*_*3*_ = 4.2298x10^-4^. While it was detailed that such parameters were chosen to physically reflect the RBC relaxation properties (namely, 5 s^-1^ corresponding to a value of 200 ms for the human RBC relaxation time), there was no experimental validation of deformation data produced using these parameters [18].

At a high level, a comparison of the current approach to that in Pauli *et al.* by analyzing the order of magnitude of the source and sink terms in Eq. 3 can be made**.** For example, at 10,000 s^-1^, the ratio between the terms scaling the deformation sink (*f*_*1*_/$\tau$ ~780 s^-1^) and the deformation source (*f*_*2*_ = 0.78) is $o\left( {10^{2} } \right)$ using the constitutive parameters in this study, whereas in Pauli *et al.*, this relationship takes $o\left( {10^{4} } \right)$. This demonstrates a difference in the numerical restorative source by two orders of magnitude. Further, the flow’s rotational component (***W***) is scaled by *f*_*3*_ = 4.2298x10^-4^ in Pauli *et al.*, contributing as an additional deformation source. These differences highlight the changes made to the model’s constitutive parameters in this study in order to reproduce experimental deformation data with reasonable MAE.

### Future Work in Complex Flows Characteristic of MCSDs

**Fig. 9:**
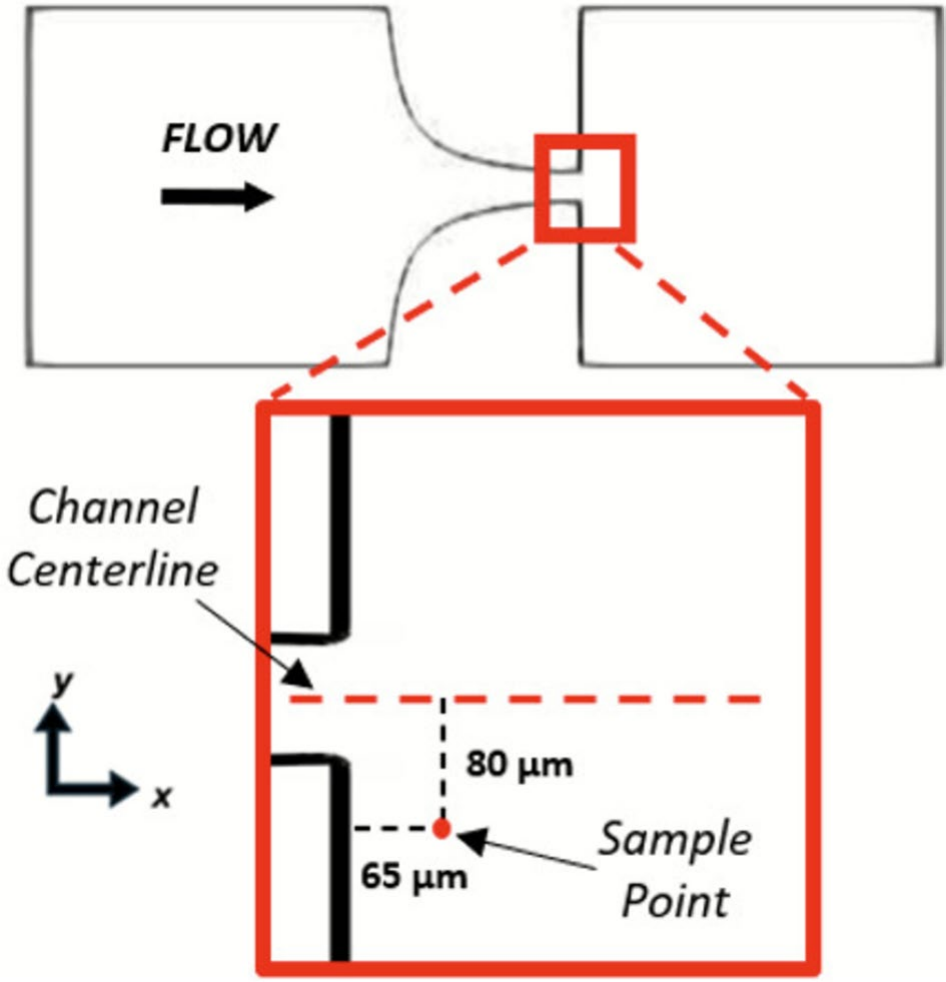
Location of additional sampling point for deformation analysis. *In silico* droplet and *in vitro* RBC deformation values were quantified at the sampling point shown in the hyperbolic converging microchannel’s sudden expansion. The point is 65 μm downstream from the channel’s throat and 80 µm from the channel’s centerline

The purpose of this study was to evaluate the model in canonical scenarios (uniaxial tension and one-dimensional shear); however, more complex scenarios should be evaluated to demonstrate the applicability of the technique to more flows characteristic of MCSDs. Although the hyperbolic converging microchannel provides a spatially varying environment akin to MCSDs along its centerline, the deformation mode remains simple and uniaxial. Therefore, additional analysis was completed in the hyperbolic converging microchannel in a sampling location with higher complexity in the flow. A location 65 µm downstream from the contraction’s throat and 80 µm offset from the channel’s centerline was sampled *in vitro* and *in silico* (Fig. 9). The sampling point is in a region with high spatial variability in the velocity and velocity gradient tensor, such that deformation is multidimensional and non-constant along streamlines.

The DI was evaluated at this location at 400 µL/min for 300 RBCs over 6 experiments in both viscosity conditions (in Plane 1 only for brevity). The velocity gradient at this location takes the following form: $$\nabla u=\left[\begin{array}{ccc}-\mathrm{6,418}& \mathrm{8,281}& 0\\ \mathrm{12,573}& \mathrm{5,893}& 0\\ 0& 0& 528\end{array}\right]$$. This provides a complex environment akin to MCSDs where flow is supraphysiological and has components of rotation, shear, and compression. The droplet DI falls within the SEM of the experimental results in both viscosity cases at this location (Table 4), showing the capability of the model to expand to more complex scenarios. The MAE value is 0.14 for *α* = 1.44 and 0.13 for *α* = 3, reflecting variability in the RBCs’ responses when the flow is multidimensional and contains both shear and extensional components. Despite this variability, these data provide insight that the model can be expanded to more complex cases, where the RBCs must adapt over a small spatial distance to varying strain rates and non-isolated flow types.

**Table 4:** *In silico* droplet and *in vitro* RBC deformation index values at the sample point, noted in Fig. 9, in the hyperbolic converging microchannel sudden expansion. *In vitro* data are reported as mean ± standard error of the mean.

Viscosity ratio	*In vitro* RBC DI	*In Silico* droplet DI	MAE
1.44	0.2 ± 0.07	0.18	0.14
3	0.16 ± 0.09	0.15	0.13

## Limitations

This study only considered 0.5% HCT, as the numerical model assumes a dilute solution of droplets that do not interact with one another. However, at physiological HCT, both the flow and viscous interaction between neighboring RBCs will contribute to deformation and RBC behavior. Work is ongoing to calibrate this model for physiological HCT, whereby an effective viscosity ratio can be used to model the effect of RBC:RBC interactions in a whole blood solution, rather than directly resolving these forces. This will maintain computational feasibility while also capturing the physical effect of viscous interactions leading to membrane deformation in whole blood. RBC dynamics will also impact the accuracy of the current approach at physiological HCT values. For example, RBCs in dense suspensions can potentially affect the flow, thereby altering deformation behavior. However, the current approach is based on a continuum assumption of suspended droplets that will not influence the flow field. Previous work has shown that in square microfluidic channels on the order of 100 $\upmu$m in width, a continuum assumption of blood can be used, as velocity profiles of blood at dense HCT values matched the analytical solution of a pure fluid in the channel [41]. An additional limitation is that the model is restricted to flows where RBCs are ellipsoidal. Therefore, flows where RBCs assume distinct non-ellipsoidal shapes (i.e., parachuting in capillary flows, slipper formation, multi-lobe and other transitional membrane configurations) are out of scope for the current approach.

Despite these limitations, the current work provides a solid foundation to apply to MCSDs, as we use supraphysiological strain rates and physiologically relevant viscosity conditions. We have also shown that the model can be extended to a more complex flow case alike to MCSD flows (Fig. 9). The model produces reasonable MAE in shear and extensional flows and therefore is deemed applicable for these investigations moving forward.

Although the present study has only considered the deformation of *in vitro* RBCs and *in silico* droplets, the long-term goal is to extend the framework to predict hemolysis in MCSDs. Broadly, this will be achieved by quantifying membrane areal strain and subsequent hemoglobin release. The RBC membrane will lyse after exposure to areal strain of ~ 6%; the convergent series for the surface area of a triaxial ellipsoid can be used to quantify areal strain based on droplet deformation results [44]. The release of hemoglobin into the flow upon RBC lysis can then be modeled with unsteady advection-diffusion equations. Additionally, this study only considered single-pass microfluidic systems. However, cyclic or repeated deformation will impact RBCs’ mechanics in MCSDs and can lead to stiffening and/or lysis. In future work, the time history of seeded particles can be tracked and sub-hemolytic damage due to cyclic or repeated exposures quantified *in silico*. These data can be validated with published *in vitro* cyclic loading data applied to the RBC membrane. The highlighted importance of the current study is capturing human RBC deformation accurately so that the areal strain and subsequent hemolysis predictions are accurate in future model development.

Finally, the *in vitro* experiments in this study evaluated 3-dimensional deformation of RBCs using a pseudo-3-dimensional approach, whereby two separate visualization planes were used to capture the cells’ 3-dimensional deformations. However, the approach used has a high fidelity for capturing 3D deformation data of the human RBCs and serves as a foundation of deformation data for the model’s calibration.

## Concluding Remarks

A droplet deformation model was modified and tested at supraphysiological strain rates using *in vitro* human RBC deformation data in this study. The highlighted *in vitro* novelty in this work is the inclusion of multiple flow types (shear, extensional) and a 3-dimensional imaging technique in the *in vitro* experiments. The approach is most applicable toward high strain rate flows, specifically those relevant to MCSDs with viscosities near that of whole blood. The model replicates human RBC flow-induced deformation behaviors, including the difference between deformation in shear and extensional flows and threshold RBC deformation values. This modeling framework is computationally feasible for application to the macroscale, complex fluid dynamic environments characteristic to MCSDs.

## Supplementary Information

Below is the link to the electronic supplementary material.

Supplementary file1 (MP4 1023 KB)

Supplementary file1 (DOCX 421 KB)
