# Supplementary material for: Modeling Red Blood Cell Deformation at Supraphysiological Strain Rates Using a Droplet Framework
Source: Ann Biomed Eng. 2026 Jan 29;54(5):1345–64. doi: 10.1007/s10439-026-04000-4 (PMC13091855; doi:10.1007/s10439-026-04000-4)
Supplement: Supplementary file 2 — Supplementary file1 (DOCX 421 KB) [file 10439_2026_4000_MOESM2_ESM.docx]

***Supplementary Information***

***S.1***

***Code Verification:*** A verification study was conducted to confirm accurate numerical implementation of the droplet deformation model. Published analytical solutions for droplet deformation in arbitrary shear, planar extensional, and uniaxial extensional flows that use the original expressions for *f_1_* and *f_2_* were used for the verification study [17]. The tensorial structure of the velocity gradient in each case is as follows: one non-zero component for simple shear flow ($\frac{{\partial u}_{1}}{{\partial x}_{2}}$), two non-zero components for planar hyperbolic flow ($\frac{{\partial u}_{1}}{{\partial x}_{1}}=-\frac{{\partial u}_{2}}{{\partial x}_{2}}$), and three non-zero components for uniaxial extensional flow ($\frac{{\partial u}_{1}}{{\partial x}_{1}}=-2\frac{{\partial u}_{2}}{{\partial x}_{2}}=-2\frac{{\partial u}_{3}}{{\partial x}_{3}}$). Time step refinement was used to evaluate the accuracy of the solutions in each case, using time steps from an order of 10^-6^ to 10^-8^ s. The numerical errors on the eigenvalues of ***S*** ${(\lambda}_{i}$) are shown in **Figure S.1.1** for *Ca* = 0.2175. Error decays with time step refinement below 10^-4^ in all cases.

***
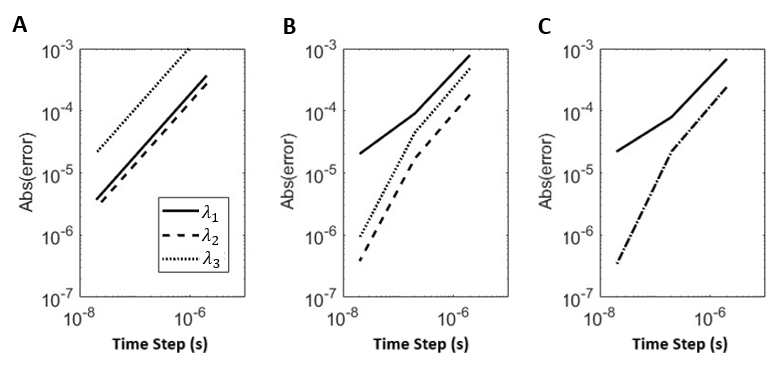
***

**Figure S.1.1. Numerical verification study results.** Three flow types are evaluated using time step refinement for code verification: **(A)** Simple Shear, **(B)** Planar Hyperbolic, and **(C)** Uniaxial Extensional Flow. The numerical error of solutions (the droplet eigenvalues, $\lambda_{i}$) at *Ca* = 0.2175 is plotted for each flow type.

***S.2***

***Model Constitutive Parameter Modifications:*** The original form of *f_1_* from Maffettone and Minale’s droplet deformation model is shown in **Eqn S.2.1**[17]. There are two expressions presented for *f_2_*: one which varies only with $\alpha$ (**Eqn S.2.2**), and one that varies with both $\alpha$ and *Ca* to capture deformation in high *Ca* flows (**Eqn S.2.3**)[17].

| $f_{1}=\frac{40(\alpha+1)}{(2\alpha+3)(19\alpha+16)}$**(S.2.1)** |
| --- |
| $f_{2}=\frac{5}{2\alpha+3}$ **(S.2.2)** |

$f_{2}=\frac{5}{2\alpha+3}+ \frac{3{Ca}^{2}}{2+6{Ca}^{2}}$ **(S.2.3)**

Deformation index comparisons using **Eqn S.2.1** for *f_1_* and **Eqn S.2.2** or **Eqn S.2.3** for *f_2_* in shear flow (Plane 1 displayed only for brevity) are shown in **Figure S.2.1**. Data are presented in **Figure S.2.1** with *in vitro* deformation results from this study. Deformation results using **Eqn S.2.2** do not have accurate strain-rate-varying behavior, while results using *f_2_* as a function of *Ca* (**Eqn S.2.3)** predict droplet breakup prematurely.

Deformation index comparisons using **Eqn S.2.1** for *f_1_* and **Eqn S.2.2** for *f_2_* in extensional flow at 1,330 s^-1^ (Plane 1 displayed only for brevity) are shown in **Figure S.2.2**. Data are presented in **Figure S.2.2** with *in vitro* deformation results from this study. Data are only shown using **Eqn S.2.2** for *f_2_* as droplet breakup was predicted in any case using **Eqn S.2.3**. The numerical behaviors shown in **Figures S.2.1** and **S.2.2** are generally preserved regardless of the droplet relaxation time.

**
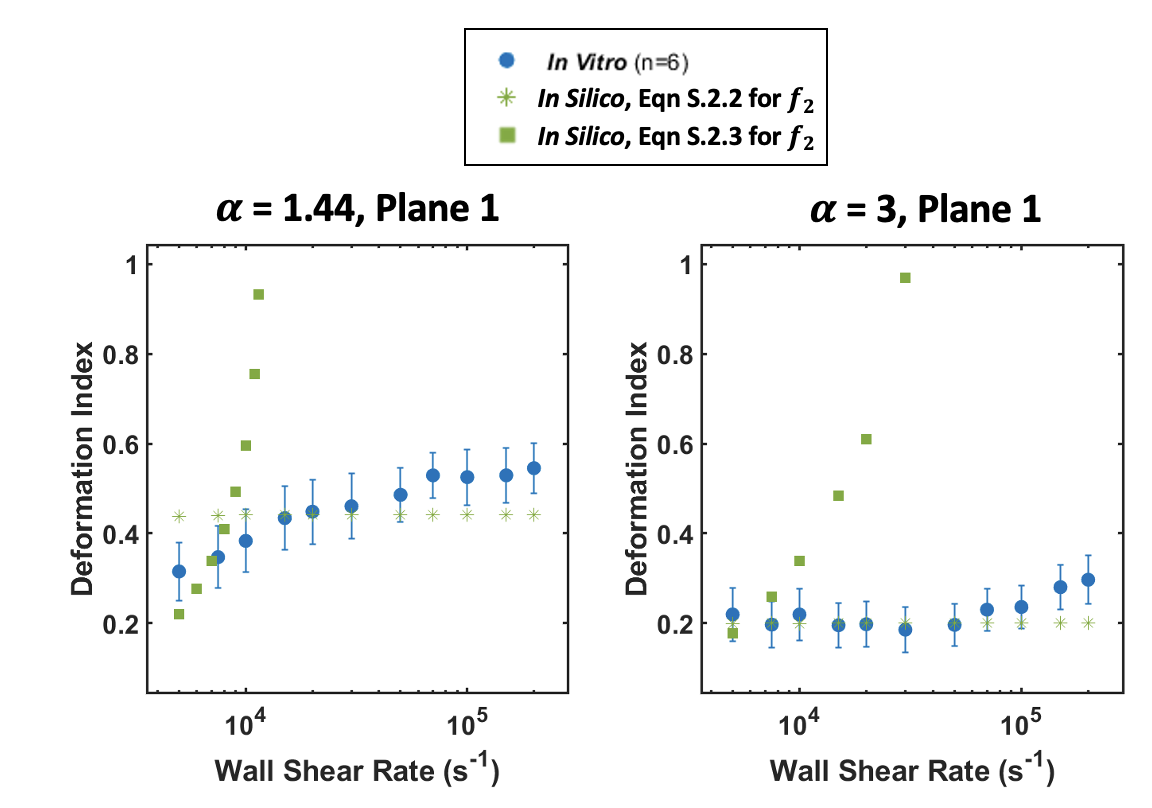
**

**Figure S.2.1.** *In vitro* and numerical shear flow results using **Eqn S.2.1** for *f_1_* and **Eqn S.2.2** or **Eqn S.2.3** for *f_2_* at two viscosity conditions ($\alpha=1.44$ and $\alpha=3$). Data are shown in Plane 1 only for brevity.


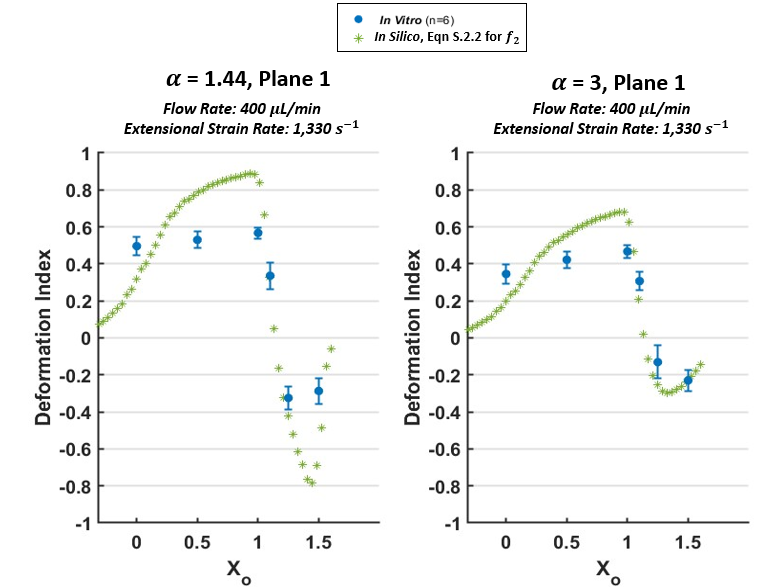


**Figure S.2.2.** *In vitro* and numerical extensional flow results using **Eqn S.2.1** for *f_1_* and **Eqn S.2.2** for *f_2_* at two viscosity conditions ($\alpha=1.44$ and $\alpha=3$). Data are shown in Plane 1 only for brevity.

These data demonstrated that *f_2_* required higher asymptotic limits to match the threshold deformations accurately in shear but also required a restorative effect to not produce premature droplet breakup at high strains and/or in extensional flows. Therefore, the coefficients on *f_2_* were modified to alter its asymptotic limits, while *f_1_* was modified to increase with increasing tensor magnitude to preserve droplet stability in high *Ca* flows. A hyperbolic tangent function was used to produce this increasing behavior for *f_1_* so that it remained bounded as physically required by the droplet deformation model. The modifications implemented to both parameters are shown in **Figure S.2.3**.

**
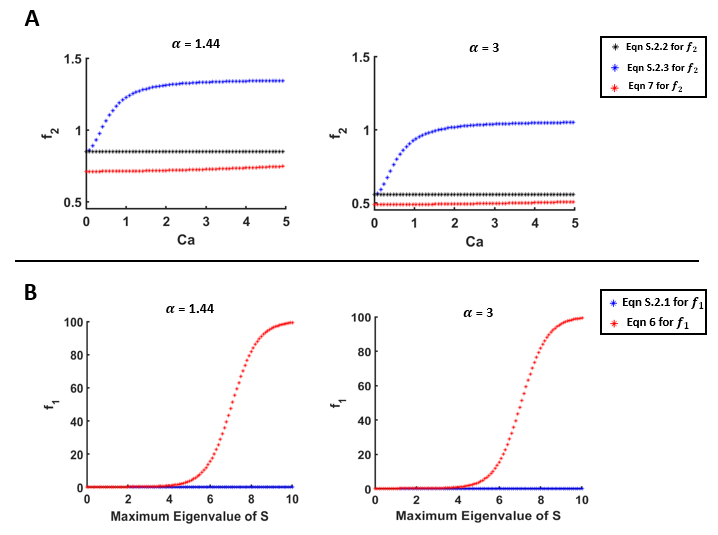
Figure S.2.3. Dependence of *f_2_* on *Ca* and dependence of *f_1_* on the maximum eigenvalue of the morphology tensor. A:** *f_2_* vs. *Ca* for both viscosity conditions, where the final expression for *f_2_* used in this study is shown with red markers. **B:** *f_1_* vs. the maximum eigenvalue of the morphology tensor, where the final expression for *f_1_* used in this study is shown with the red markers.

We note here for clarity that $f_{1}$and $f_{2}$ are dimensionless numerical coefficients that do not correspond directly to a single RBC mechanical property. Instead, they are the mathematical result of a first-order expansion of **Eqn 3** which can be found in [31] and their values are restricted only by the physical requirements of the model (i.e., the parameters are non-negative and bounded). We therefore selected $f_{1}$and $f_{2}$ to ensure *(1)* stability of the morphology tensor and *(2)* recovery of experimentally measured asymptotic deformation limits (DI ≈0.6 at supraphysiological strain rates). Further, with these modifications implemented, droplet deformation remains physical according to the requirements presented by Maffettone and Minale: deformation varies linearly with *Ca* in low *Ca* flows and droplet volume is preserved at any *Ca* [17].

We also note here that the combination of all constitutive parameters ($\tau{, f}_{1}$, and $f_{2}$) produce the *in silico* deformation behavior(s). As there is a wide range of experimentally determined interfacial tension values for the RBC membrane [32-36], $\tau$ and *Ca* (and subsequently, $f_{1}$, and $f_{2}$) will be affected significantly by selecting a different interfacial tension value within this range. If a different value of interfacial tension is selected, the equations for$f_{1}$ and $f_{2}$ will need to be updated to reflect the differing relaxation dynamics.

***S.3***

***Computational Refinement:*** The *in silico* solutions were verified to be independent of the computational mesh used. Three meshes were investigated for the simple shear channels: structured hex elements of 50,000, 112,000 and 180,000 cells were made with OpenFOAM’s (version 7) blockMesh utility. The grid was refined near the walls in the shear channels to capture high velocity gradients. The hyperbolic converging microchannel was meshed using Fidelity Pointwise 2023.1.1 (Cadence Design Systems, San Jose, CA), and the grid was refined near the channel walls and corners at its throat. Grids comprising of 58,000, 272,000, and 539,000 structured hex elements were used. The minimum and maximum flow rates were used for each flow type for the mesh independence study (manuscript text **Table 2**). The fluid mechanics were first deemed mesh independent using OpenFOAM v7 by comparing the velocity, pressure, and velocity gradient values at the regions of interest in the channels. For shear flow there was ~8% difference between the coarse and medium meshes, and a 1-2.2% difference between the medium and fine meshes for the flow parameters in both flow conditions. In extensional flow, there was a 6% difference in flow parameters between coarse and medium meshes and less than 2% differences between the medium and fine meshes. The deformation model was then run using each mesh. For shear flow, there was <5% difference in deformation results between the coarse and medium meshes and <1% difference in deformation results between the medium and fine meshes. For extensional flow, 6% difference in deformation existed between the coarse and medium meshes, while there was <1% difference in deformation between the medium and fine meshes. Therefore, the medium meshes were used for all *in silico* experiments.

***S.4***

***Mean Absolute Error Values for In Silico Deformation Predictions:*** The mean absolute error (MAE, **Eqn 13**) was calculated for *in silico* DI data in both Plane 1 and Plane 2 for α = 3 and α = 1.44 across all shear and extensional flow conditions. The results from this analysis are shown in **Table S.4.1**, **Table S.4.2**, and **Table S.4.3**.

**Table S.4.1** shows the MAE values across the 11 shear flow conditions in both Plane 1 and Plane 2 for both viscosity conditions (α = 1.44 and α = 3). **Table S.4.2** shows the MAE values across the 3 extensional flow conditions at each of the 6 imaging locations (X_o_ = 0, 0.5, 1, 1.1, 1.25, and 1.5) in both Plane 1 and Plane 2 for α = 1.44. **Table S.4.3** shows the MAE values across the 3 extensional flow conditions at each of the 6 imaging locations (X_o_ = 0, 0.5, 1, 1.1, 1.25, and 1.5) in both Plane 1 and Plane 2 for α = 3.

**Table S.4.1.** MAE values of the *in silico* DI prediction compared with *in vitro* DI values across 11 shear flow conditions in two visualization planes (Plane 1, Plane 2) and two viscosity conditions (α = 1.44 and α = 3).

|  |  | **α = 1.44** | | **α = 3** | |
| --- | --- | --- | --- | --- | --- |
|  |  | **Plane 1** | **Plane 2** | **Plane 1** | **Plane 2** |
| **Wall Shear Rate,** ${\dot{\boldsymbol{\gamma}}}_{\boldsymbol{Shear}}$ **(s^-1^)** | **5,000** | 0.12 | 0.09 | 0.09 | 0.10 |
|  | **7,500** | 0.12 | 0.08 | 0.10 | 0.09 |
|  | **10,000** | 0.15 | 0.08 | 0.11 | 0.09 |
|  | **15,000** | 0.14 | 0.07 | 0.09 | 0.10 |
|  | **20,000** | 0.15 | 0.06 | 0.09 | 0.10 |
|  | **30,000** | 0.14 | 0.06 | 0.10 | 0.09 |
|  | **50,000** | 0.11 | 0.05 | 0.09 | 0.09 |
|  | **70,000** | 0.09 | 0.06 | 0.09 | 0.11 |
|  | **100,000** | 0.12 | 0.06 | 0.10 | 0.10 |
|  | **150,000** | 0.11 | 0.05 | 0.10 | 0.09 |
|  | **200,000** | 0.10 | 0.06 | 0.08 | 0.09 |

**Table S.4.2.** MAE values of the *in silico* DI value compared with *in vitro* deformation data at each of the 6 imaging locations in the hyperbolic converging microchannel (X_o_ = 0, 0.5, 1, 1.1, 1.25, and 1.5) in both visualization planes (Plane 1, Plane 2) for α = 1.44.

|  |  | **Plane 1** | | | | | | **Plane 2** | | | | | |
| --- | --- | --- | --- | --- | --- | --- | --- | --- | --- | --- | --- | --- | --- |
| **X_o_** | | **0** | **0.5** | **1** | **1.1** | **1.25** | **1.5** | **0** | **0.5** | **1** | **1.1** | **1.25** | **1.5** |
| **Extensional Strain Rate, ~2***${\dot{\boldsymbol{\gamma}}}_{\boldsymbol{Extension}}$ **(s^-1^)** | **330** | 0.17 | 0.15 | 0.12 | 0.10 | 0.10 | 0.19 | 0.17 | 0.13 | 0.14 | 0.11 | 0.11 | 0.17 |
|  | **670** | 0.17 | 0.10 | 0.09 | 0.10 | 0.12 | 0.15 | 0.17 | 0.10 | 0.11 | 0.16 | 0.12 | 0.16 |
|  | **1,330** | 0.13 | 0.09 | 0.06 | 0.09 | 0.13 | 0.08 | 0.11 | 0.12 | 0.11 | 0.11 | 0.14 | 0.08 |

**Table S.4.3.** MAE values of the *in silico* DI value compared with *in vitro* deformation data at each of the 6 imaging locations in the hyperbolic converging microchannel (X_o_ = 0, 0.5, 1, 1.1, 1.25, and 1.5) in both visualization planes (Plane 1, Plane 2) for α = 3.

|  |  | **Plane 1** | | | | | | **Plane 2** | | | | | |
| --- | --- | --- | --- | --- | --- | --- | --- | --- | --- | --- | --- | --- | --- |
| **X_o_** | | **0** | **0.5** | **1** | **1.1** | **1.25** | **1.5** | **0** | **0.5** | **1** | **1.1** | **1.25** | **1.5** |
| **Extensional Strain Rate, ~2***${\dot{\boldsymbol{\gamma}}}_{\boldsymbol{Extension}}$ **(s^-1^)** | **330** | 0.19 | 0.17 | 0.17 | 0.08 | 0.09 | 0.18 | 0.15 | 0.12 | 0.13 | 0.10 | 0.12 | 0.18 |
|  | **670** | 0.20 | 0.11 | 0.09 | 0.10 | 0.07 | 0.13 | 0.14 | 0.08 | 0.08 | 0.10 | 0.09 | 0.15 |
|  | **1,330** | 0.17 | 0.09 | 0.07 | 0.22 | 0.08 | 0.05 | 0.17 | 0.10 | 0.10 | 0.22 | 0.13 | 0.08 |

***S.5***

***Inter- and Intra-Donor RBC Deformation Variability***: To evaluate the per-donor variance in *in vitro* DI measurements, intraclass correlation coefficients (ICCs**, Eqn S.5.1**) were quantified across the conditions tested in this study. This method has been used in previous studies to evaluate inter- and intra- donor blood measurement variability [40].

Briefly, the ICC is a ratio between the inter-donor DI variance ($\sigma_{Between Donors}^{2}$) and the combined sum of inter-donor and average intra-donor DI variance ($\sigma_{Between Donors}^{2}, \sigma_{Within Donors}^{2}$, respectively). This analysis was completed for a subset of the experimental conditions tested in our study for brevity.

$ICC= \frac{\sigma_{Between Donors}^{2}}{\sigma_{Between Donors}^{2}+\sigma_{Within Donors}^{2}}$ **(S.5.1)**

ICC values in shear flow for 5,000, 20,000, 100,000, and 200,000 s^-1^ are shown in **Table S.5.1** for both visualization planes (Pane 1, Plane 2) and both viscosity conditions (α=1.44, α=3). The shear flow ICC values range from 0.01 – 0.34, indicating that 1-34% of the total variability in shear flow-induced deformation data is attributed inter-donor RBC variability.

**Table S.5.1.** ICC values across 11 shear flow conditions for two visualization planes (Plane 1, Plane 2) and two viscosity conditions (α=1.44 and α=3).

|  |  | **α=1.44** | | **α=3** | |
| --- | --- | --- | --- | --- | --- |
|  |  | **Plane 1** | **Plane 2** | **Plane 1** | **Plane 2** |
| **Shear Rate (s^-1^)** | **5,000** | 0.30 | 0.05 | 0.34 | 0.01 |
|  | **20,000** | 0.23 | 0.03 | 0.19 | 0.08 |
|  | **100,000** | 0.03 | 0.02 | 0.05 | 0.1 |
|  | **200,000** | 0.08 | 0.01 | 0.08 | 0.05 |

ICC values in extensional flow at X_o_ = 1 (peak extensional strain) are shown for the three flow conditions tested (300, 670, 1,300 s^-1^) in both planes (Plane 1, Plane 2) for both viscosity conditions (α=1.44, α=3) in **Table S.5.2**. The extensional flow ICC values at X_o_ = 1 range from 0.03 – 0.28, indicating that 3-28% of the total variability in extensional flow-induced deformation data is attributed inter-donor RBC variability.

**Table S.5.2.** ICC values across 3 extensional flow conditions at X_o_ = 1 for two visualization planes (Plane 1, Plane 2) and two viscosity conditions (α=1.44 and α=3).

|  |  | **α=1.44** | | **α=3** | |
| --- | --- | --- | --- | --- | --- |
|  |  | **Plane 1** | **Plane 2** | **Plane 1** | **Plane 2** |
| **Extensional Strain Rate (s^-1^)** | **330** | 0.14 | 0.16 | 0.04 | 0.15 |
|  | **670** | 0.20 | 0.19 | 0.09 | 0.09 |
|  | **1,330** | 0.10 | 0.28 | 0.05 | 0.03 |

Further, ICC values in extensional flow at X_o_ = 1.5 are shown for both planes (Plane 1, Plane 2) and both viscosity conditions (α=1.44, α=3) in **Table S.5.3**. The extensional flow ICC values at X_o_ = 1.5 range from 0.06 – 0.35, indicating that 6-35% of the total variability in these flow-induced deformation data is attributed inter-donor RBC variability.

**Table S.5.3.** ICC values across 3 extensional flow conditions at X_o_ = 1.5 for two visualization planes (Plane 1, Plane 2) and two viscosity conditions (α=1.44 and α=**3**).

|  |  | **α=1.44** | | **α=3** | |
| --- | --- | --- | --- | --- | --- |
|  |  | **Plane 1** | **Plane 2** | **Plane 1** | **Plane 2** |
| **Extensional Strain Rate (s^-1^)** | **330** | 0.31 | 0.08 | 0.35 | 0.06 |
|  | **670** | 0.15 | 0.33 | 0.25 | 0.17 |
|  | **1,330** | 0.22 | 0.20 | 0.24 | 0.06 |
